# Prognostic Implications of Programmed Cell Death Ligand 1 Expression, Cluster of Differentiation 8‐Positive T‐Cell Infiltration, and Related Immunophenotypes in Invasive Mucinous Adenocarcinoma of the Lung: A Multicenter Study

**DOI:** 10.1002/mco2.70710

**Published:** 2026-04-05

**Authors:** Guochao Zhang, Chao Zheng, Jia Jia, Xingchen Li, Lide Wang, Long Zhang, Yuzhuo Zhang, Meng Yue, Shuangping Zhang, Yueping Liu, Liyan Xue, Qi Xue, Jie He

**Affiliations:** ^1^ Department of Thoracic Surgery National Cancer Center/National Clinical Research Center for Cancer/Cancer Hospital Chinese Academy of Medical Sciences and Peking Union Medical College Beijing China; ^2^ Department of Cancer Prevention National Cancer Center/National Clinical Research Center for Cancer/Cancer Hospital Chinese Academy of Medical Sciences and Peking Union Medical College Beijing China; ^3^ Department of Pathology National Cancer Center/ National Clinical Research Center For Cancer/Cancer Hospital Chinese Academy of Medical Sciences and Peking Union Medical College Beijing China; ^4^ Department of Pathology Fourth Hospital of Hebei Medical University/Tumor Hospital of Hebei Province Shijiazhuang China; ^5^ Department of Thoracic Surgery Shanxi Province Cancer Hospital/Shanxi Hospital Affiliated to Cancer Hospital Chinese Academy of Medical Sciences Taiyuan China

**Keywords:** cluster of differentiation 8, immune phenotype, invasive mucinous adenocarcinoma of the lung, immune checkpoint, programmed cell death ligand 1, rare malignancy

## Abstract

The immune microenvironment of invasive mucinous adenocarcinoma of the lung (IMA), a rare and heterogeneous subtype, remains poorly characterized, limiting insights into its potential response to immunotherapy. In this multicenter study, we systematically evaluated programmed cell death ligand 1 (PD‐L1) expression (using tumor proportion score, TPS, and combined positive score, CPS) and cluster of differentiation 8‐positive (CD8^+^) tumor‐infiltrating lymphocyte (TIL) infiltration in the largest cohort to date of pathologically confirmed pure IMAs (*n* = 312), supported by single‑cell transcriptomic analysis. PD‐L1 positivity was low (TPS≥1%: 9.0%; CPS≥1: 28.5%). While PD‐L1 alone showed no prognostic significance, high CD8^+^ TIL percentage and density were independent, favorable prognostic factors for relapse‐free survival, particularly in patients not receiving adjuvant therapy. By integrating TPS and CD8^+^ TIL percentage, we established a novel four‐category immune phenotype classification that identified a distinct subgroup (Type IV: PD‐L1^+^/CD8^+^) with significantly better outcomes. Preliminary analysis of 20 patients who received immune checkpoint inhibitors suggested that Type IV patients may derive greater clinical benefit. Single‐cell RNA sequencing analyses revealed a paucity of effector CD8^+^ T cells in IMA. This work defines the unique immune landscape of IMA, introduces a clinically relevant immune phenotyping framework with prognostic and predictive potential, and provides a rationale for future immunotherapeutic strategies in this rare malignancy.

## Graphical abstract

An immune phenotype classification for invasive mucinous adenocarcinoma (IMA) was established based on PD‐L1 expression and CD8^+^ T‐cell infiltration in a large multicenter cohort (n = 312), supplemented by an immunotherapy cohort (*n* = 20) and single‐cell RNA sequencing analysis. PD‐L1 positively correlated with CD8^+^ TILs, while higher CD8^+^ TILs independently predicted improved relapse‐free survival. The four‐category immunophenotype model effectively stratified patient prognosis and showed a preliminary association with immunotherapy response. Single‐cell analysis revealed a paucity of effector CD8^+^ T cells in IMA, providing mechanistic insights into its unique immune microenvironment.

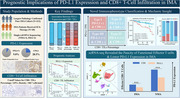

## 1 Introduction

Invasive mucinous adenocarcinoma of the lung (IMA)is a rare and distinct histological subtype of non–small cell lung cancer (NSCLC) [1]. Characterized by aggressive local invasion, higher rates of multifocal and metastatic disease, and a generally poorer prognosis than other NSCLCs, IMA poses significant therapeutic challenges in oncology [2]. A hallmark feature of IMA, the abundant production of mucin, not only contributes to its unique pathological profile but also influences its response to various treatments, including emerging immunotherapies [3].

The emergence of immune checkpoint inhibitors (ICIs), specifically those targeting programmed death‐ligand 1 (PD‐L1) and programmed death‐1 (PD‐1), has revolutionized the management of NSCLC. These therapies have shown remarkable efficacy across various NSCLC subtypes, changing the cancer treatment landscape [4, 5, 6]. However, the efficacy of ICIs is not uniform across all patients, leading to an intensified search for predictive biomarkers that can accurately predict treatment responses and guide clinical decision‐making [7].

PD‐L1 expression and cluster of differentiation 8‐positive (CD8^+^)*p* = tumor‐infiltrating lymphocyte (TIL) have emerged as significant biomarkers. PD‐L1, a critical immune checkpoint molecule, is often upregulated in tumors and immune cells and plays a pivotal role in cancer immune evasion [8]. On the other hand, the infiltration of CD8^+^ TILs signifies an active immune response against the tumor, so these cells are considered markers of tumor immunogenicity [9].

Although recent studies have provided considerable insight into the immune microenvironment of NSCLC, the specific dynamics of IMA in this context remain underexplored. This is mainly due to its rarity and distinct biological behavior, which may differ significantly from those of other NSCLC subtypes [10]. Therefore, a focused investigation into the patterns of PD‐L1 expression and CD8^+^ TIL infiltration in IMA is critical. Such research could uncover novel prognostic strategies and guide the development of tailored immunotherapeutic strategies for IMA patients [11].

Our study involves a comprehensive exploration of PD‐L1 expression and CD8^+^ TIL infiltration within IMA patient cohorts. Surgical specimens of IMA patients from various centers were systematically analyzed to elucidate the characteristics of the immune microenvironment. We aimed to elucidate the mechanisms underlying the interaction between IMA and the immune system, thereby offering substantial support for the development of personalized cancer treatment strategies.

Furthermore, we focused on investigating the correlation among PD‐L1 expression, CD8^+^ TIL infiltration, and clinical outcomes, deepening the scope and breadth of our research. This comprehensive analysis revealed the potential utility of these biomarkers in predicting patient responses to immunotherapy and in stratifying patients for tailored treatment approaches.

Through this study, we aimed to address critical gaps in the current understanding of IMA, potentially leading to the development and improvement of personalized treatment regimens, thereby enhancing the prognosis for patients with this challenging subtype.

## 2 Results

### 2.1 Baseline Characteristics

A total of 312 patients with resected IMA were included, with baseline characteristics detailed in Table S1. The majority of patients were female (59.9%), and close to half (49.0%) were over 60 years old. Approximately one‐quarter had a history of smoking (26.6%) or alcohol consumption (17.0%). As expected, IMA tended to occur more frequently in the right lung (50.3%) and lower lobe (68.3%). The vast majority of included patients had early‐stage mucinous adenocarcinoma (tumor node metastasis [TNM] stage I: 69.2%). Most (88.5%) patients underwent video‐assisted thoracoscopic surgery, and 29.8% of patients received postoperative adjuvant therapy.

### 2.2 CD8^+^ TIL Infiltration and Clinicopathological Characteristics

**FIGURE 1:**
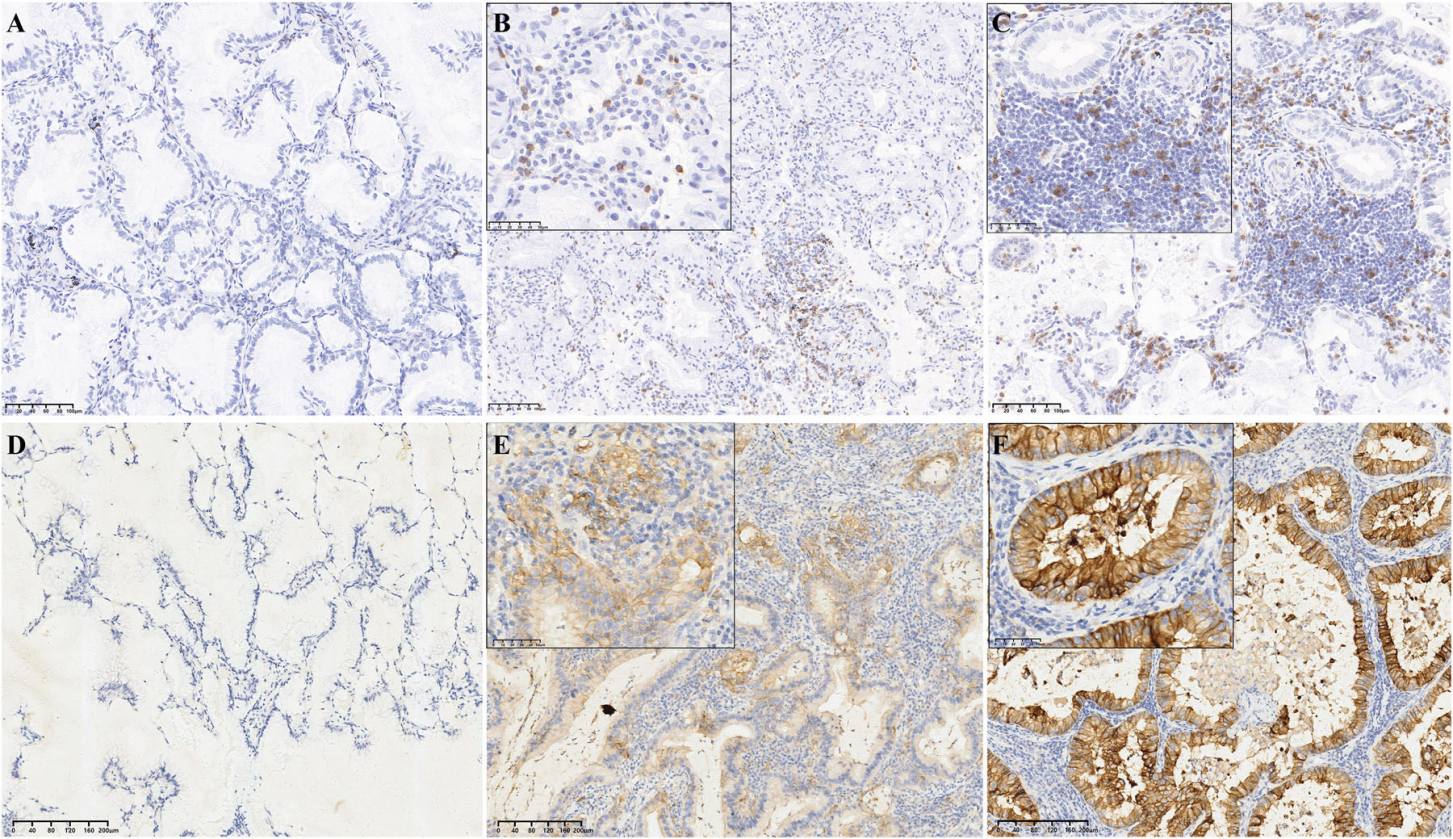
Expression of CD8 and PD‐L1 in patients with IMA (IHC staining, ×200). We demonstrated that CD8^+^ lymphocyte level differed among different cases of lung invasive mucinous adenocarcinoma (low (A), medium (B), and high levels (C)) and PD‐L1 expression (zero (D), low (E), and high expression (F)).

**FIGURE 2:**
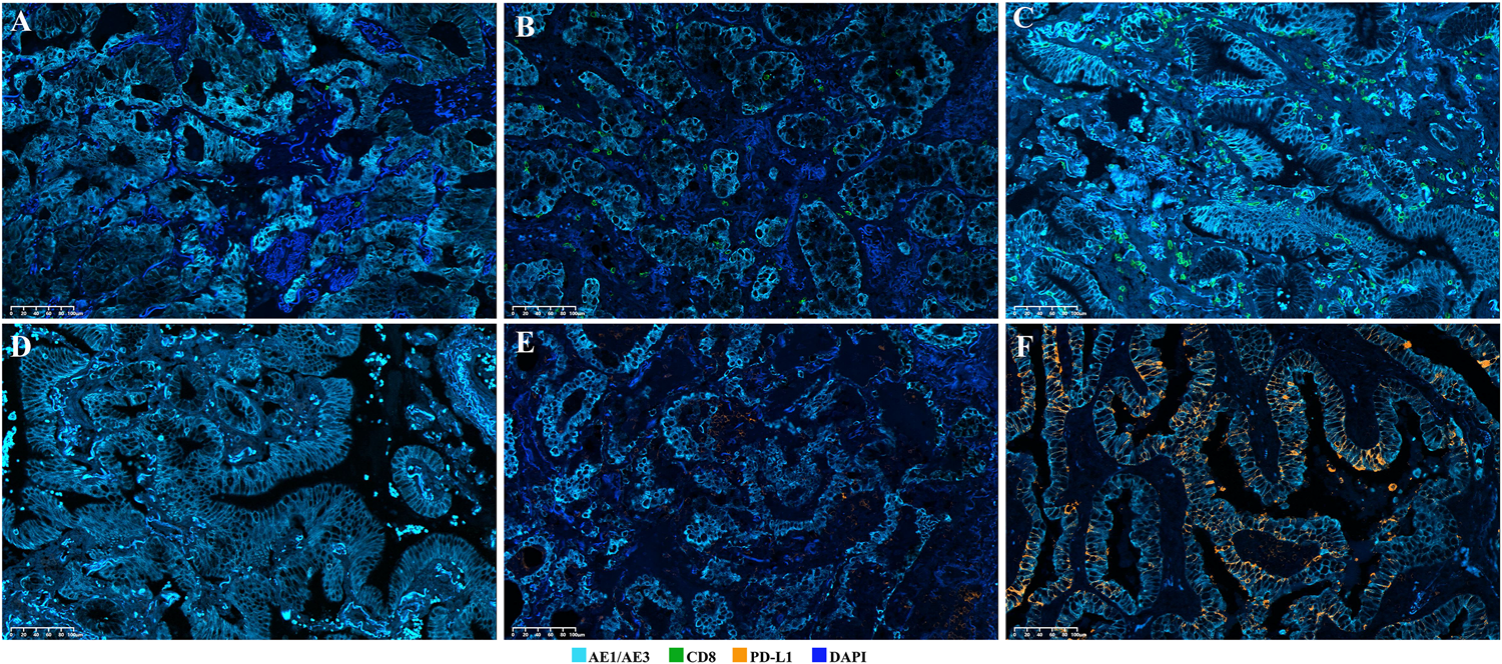
Expression of CD8 and PD‐L1 in patients with IMA (mIF staining, ×200). We labeled tumor cells with AE1/AE3 and grouped the samples according to CD8^+^ lymphocyte levels (low (A), medium (B), and high (C) levels); similarly, PD‐L1 expression (zero, low, and high) in the tumor cells is presented in the D‐F graphs.

**FIGURE 3:**
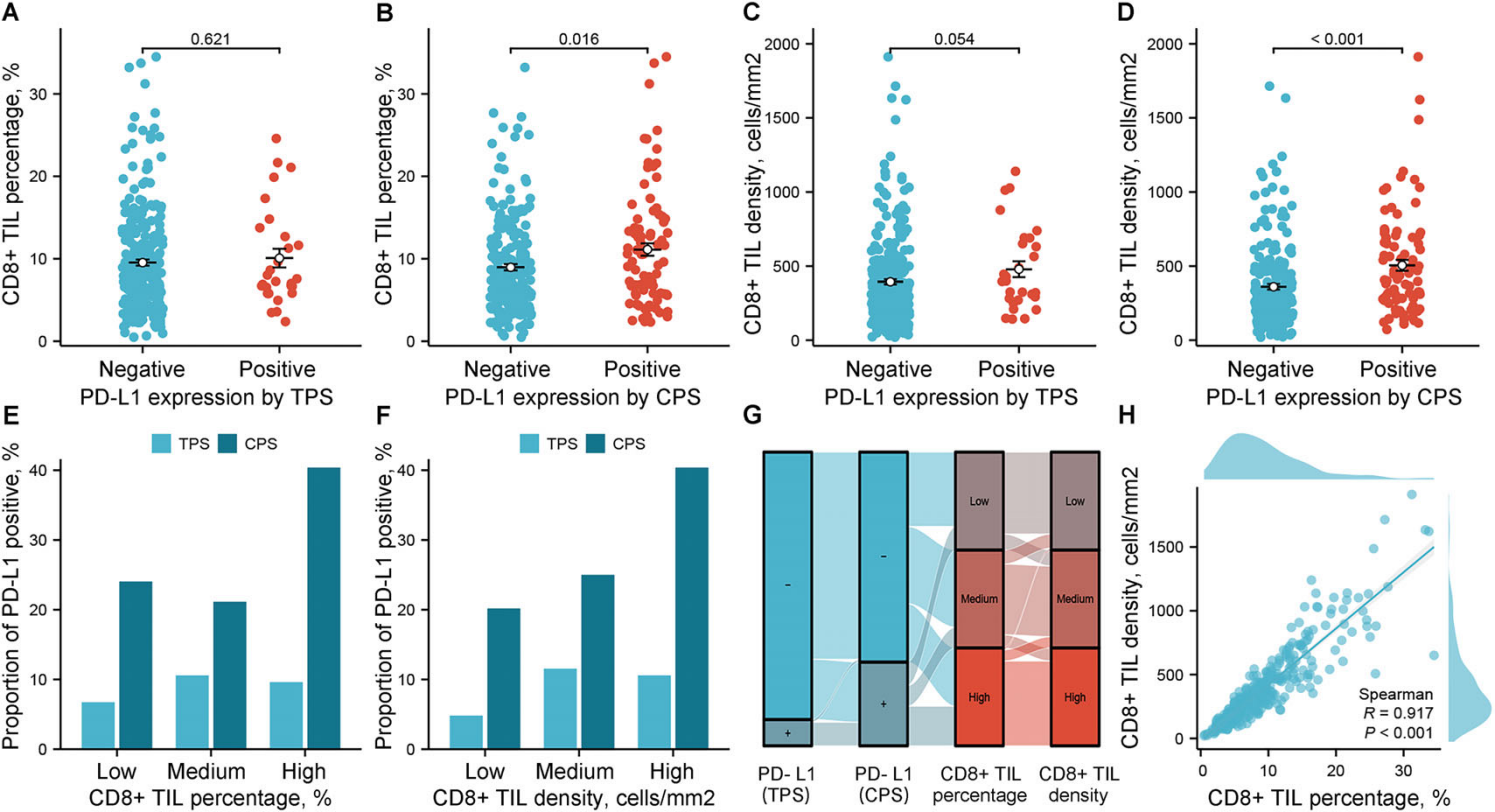
Association between CD8^+^ T‐cell infiltration and PD‐L1 expression. Panels A and B show the comparison of the percentage of CD8^+^ TILs between PD‐L1‐negative and PD‐L1‐positive samples based on different scoring criteria (TPS and CPS, respectively). Panels C and D show the differences in CD8^+^ TIL density between PD‐L1‐negative and PD‐L1‐positive samples using the same scoring criteria. Panels E and F show the comparison of the proportions of patients who were PD‐L1‐positive among patients with different percentages and densities of CD8^+^ TILs, respectively. Panel G shows the distributions of CD8^+^ T cell levels and PD‐L1 expression status in the IMA cohort. Panel H shows the scatter plot with a linear fit between the percentage and density of CD8^+^ TILs.

CD8^+^ T cells can be suppressed by PD‐L1 expression, playing a pivotal role in adaptive immune responses. Thus, their presence and characteristics are key determinants of the potential responsiveness of tumors to ICI therapies. We quantified CD8^+^ TIL infiltration in IMA by calculating the percentage and density of CD8^+^ TILs. Representative immunohistochemistry (IHC) and multiplex immunofluorescence (mIF) images of CD8^+^ T cells are presented in Figure 1A‒C and Figure 2A‒C, respectively. The median and mean percentages of CD8^+^ TILs among all patients were 8.3% (interquartile range [IQR]: 5.2–12.6%) and 9.6% (standard deviation [SD]: 6.2%), respectively. The median and mean CD8^+^ TIL densities for the IMA patients were 320.2 cells/mm^2^ (IQR: 192.8–507.6 cells/mm^2^) and 401.7 cells/mm^2^ (SD: 304.6 cells/mm^2^), respectively. A substantial linear correlation was observed between the percentage and density of CD8^+^ TILs (Spearman's test, *R* = 0.917, *p* < 0.001) (Figure 3H).

The correlations between CD8^+^ TILs and clinicopathological variables are summarized in Table 1. Based on X‐tile analyses, the optimal cutoff values for the percentage and density of CD8^+^ TILs for predicting relapse‐free survival (RFS) were 3.8% and 148.7 cells/mm^2^, respectively. The percentage of CD8^+^ TILs was significantly associated with comorbidities (*p* = 0.011), tumor location/lobe (*p* = 0.001), genetic test results *(p* = 0.004), and N stage (*p* = 0.028). CD8^+^ TIL density was significantly correlated with comorbidities (*p* = 0.003), tumor location/lobe (*p* = 0.012), genetic test results (*p* = 0.044), and T stage (*p* = 0.043). Patients with higher densities of CD8^+^ TIL were significantly associated with longer RFS and overall survival (OS). A higher CD8^+^ TIL percentage was also significantly associated with prolonged RFS, though not with OS.

**TABLE 1:** Comparison of clinicopathological characteristics and PD‐L1 expression between IMA patients with different CD8^+^ T cell infiltration levels.

	CD8^+^ TIL density, mm^2^			CD8^+^ TIL percentage, %		
	<148.7 (negative)	≥148.7(positive)		<3.8 (negative)	≥3.8 (positive)	
Characteristic	(*N* = 56, 18.0 %)	(*N* = 256, 82.0%)	*p*‐value	(*N* = 44, 14.1%)	(*N* = 268, 85.9%)	*p*‐value
Gender, *n* (%)			0.865			0.431
Male	23 (41.1)	102 (39.8)		20 (45.4)	105 (39.2)	
Female	33 (58.9)	154 (60.2)		24 (54.6)	163 (60.8)	
Age, *n* (%)			0.892			0.643
≤60 years	29 (51.8)	130 (50.8)		21 (47.7)	138 (51.5)	
>60 years	27 (48.2)	126 (49.2)		23 (52.3)	130 (48.5)	
Alcohol abuse			0.848			0.274
Yes	10 (17.9)	43 (16.8)		10 (22.7)	43 (16.0)	
No	46 (82.1)	213 (83.2)		34 (77.3)	225 (84.0)	
Smoking			0.713			0.914
Yes	16 (28.6)	67 (26.2)		12 (27.3)	71 (26.5)	
No	40 (71.4)	189 (73.8)		32 (72.7)	197 (73.5)	
Smoking index			0.625			0.626
≤400	46 (82.1)	217 (84.8)		36 (81.8)	227 (84.7)	
>400	10 (17.9)	39 (15.2)		8 (18.2)	41 (15.3)	
Family history of malignant tumor			0.461			0.427
Yes	11 (19.6)	40 (15.6)		9 (20.4)	42 (15.7)	
No	45 (80.4)	216 (84.4)		35 (79.6)	226 (84.3)	
Comorbidities			0.003			0.011
Yes	14 (25.0)	119 (46.5)		11 (25.0)	122(45.5)	
No	42 (75.0)	137 (53.5)		33 (75.0)	146 (54.5)	
Treatment modality			0.165			0.082
Surgery alone	35 (62.5)	184 (71.9)		26 (59.1)	193(72.0)	
Surgery with adjuvant therapy	21 (37.5)	72 (28.1)		18 (40.9)	75 (28.0)	
Surgical approach			0.102			0.137
VATS	46 (82.1)	230 (89.8)		36 (81.8)	240 (89.6)	
Open	10 (17.9)	26 (10.2)		(18.2)	28 (10.4)	
Laterality			0.155			0.209
Left	23 (41.1)	132 (51.6)		18 (40.9)	137 (51.1)	
Right	33 (58.9)	124 (48.4)		26 (59.1)	131 (48.9)	
Lobe			0.012			0.001
Upper	21 (37.5)	55 (21.5)		21 (47.7)	55 (20.5)	
Middle	1 (1.8)	15 (5.9)		2 (4.6)	14 (5.2)	
Lower	31 (55.4)	182 (71.1)		19 (43.1)	194 (72.4)	
Overlapping	3 (5.4)	4 (1.6)		2 (4.6)	5 (1.9)	
Tumor size, cm			0.618			0.551
≤3	33 (58.9)	160 (62.5)		29 (65.9)	164 (61.2)	
>3	23 (41.1)	96 (37.5)		15 (34.1)	104 (38.8)	
Genetic test			0.044			0.004
NA	22 (39.3)	94 (36.7)		15 (34.1)	104 (37.7)	
None	18 (32.2)	64 (25.0)		16 (36.4)	66 (24.6)	
KRAS mutation	7 (12.5)	78 (30.5)		4 (9.1)	81 (30.2)	
EGFR mutation	4 (7.1)	9 (3.5)		4 (9.1)	9 (3.4)	
Others	5 (8.9)	11 (4.3)		5 (11.3)	11 (4.1)	
T stage			0.415			0.363
T1‐2	45 (80.4)	217 (84.8)		39 (88.6)	223 (83.2)	
T3‐4	11 (19.6)	39 (15.2)		5 (11.4)	45 (16.8)	
N stage			0.065			0.028
N0	46 (82.1)	232 (90.6)		35 (79.6)	243 (90.7)	
N1‐2	10 (17.9)	24 (9.4)		9 (20.4)	25 (9.3)	
TNM stage			0.461			0.933
I/II	45 (80.4)	216 (84.4)		37 (84.1)	224 (83.6)	
III	11 (19.6)	40 (15.6)		7 (15.9)	44 (16.4)	
PD‐L1 expression by TPS, *n* (%)			0.296			0.589
Negative (<1%)	53 (94.6)	231 (90.2)		41 (93.2)	243 (90.7)	
Positive (≥1%)	3 (5.4)	25 (9.8)		3 (6.8)	25 (9.3)	
PD‐L1 expression by CPS, *n* (%)			0.023			0.101
Negative (<1)	47 (83.9)	176 (68.8)		36 (81.8)	187 (69.8)	
Positive (≥1)	9 (16.1)	80 (31.2)		8 (18.2)	81 (30.2)	
Overall survival, *n* (%)			0.016^*^			0.230^*^
Yes	46 (82.1)	238 (93.0)		38 (86.4)	246 (91.8)	
No	10 (17.9)	18 (7.0)		6 (13.6)	22 (8.2)	
Relapse‐free survival, *n* (%)			0.004^*^			<0.001^*^
Yes	39 (69.6)	223 (87.1)		28 (63.6)	234 (87.3)	
No	17 (30.4)	33 (12.9)		16 (36.4)	34 (12.7)	

Abbreviations: CPS, combined positive score; NA, not applicable; TNM, tumor node metastasis stage; TPS, tumor positive score; VATS, video‐assisted thoracoscopic surgery.

^*^Log‐rank test.

### 2.3 PD‐L1 Expression and Clinicopathological Characteristics

When a tumor proportion score (TPS) ≥1% was used, 28 of 312 patients (9.0%) with IMA were diagnosed as PD‐L1 positive, while when a combined positive score (CPS) ≥1 was used as the criterion, 89 (28.5%) were recorded as PD‐L1‐positive. Representative IHC and mIF images of PD‐L1 staining are shown in Figure 1D‒F and Figure 2D‒F, respectively. The correlations between PD‐L1 expression and clinicopathological factors are shown in Table 2. Notably, the TPS exhibited significant association with N stage (*p* = 0.012) and TNM stage (*p* = 0.004), while the CPS showed significant correlation with age (*p* = 0.019), alcohol abuse (*p* = 0.041), surgical approach (*p* = 0.001), genetic test results (*p* = 0.001), and TNM stage (*p* = 0.029). Moreover, patients who were PD‐L1‐positive according to either the TPS or CPS did not have significantly longer RFS or OS.

**TABLE 2:** Comparison of clinicopathological characteristics and CD8^+^ T cell infiltration between IMA patients with different PD‐L1 expression levels.

	PD‐L1 expression by TPS			PD‐L1 expression by CPS		
Characteristic	<1% (negative)	≥1% (positive)	*p*‐value	<1 (negative)	≥1 (positive)	*p*‐value
	(*N* = 284, 91.0%)	(*N* = 28, 9.0%)		(*N* = 223, 71.5%)	(*N* = 89, 28.5%)	
Gender, *n* (%)			0.126			0.866
Male	110 (38.7)	15 (53.6)		90 (40.4)	35 (39.3)	
Female	174 (61.3)	13 (46.4)		133 (59.6)	54 (60.7)	
Age, *n* (%)			0.615			0.019
≤60 years	146 (51.4)	13 (46.4)		123 (55.2)	36 (40.4)	
>60 years	138 (48.6)	15 (53.6)		100 (44.8)	53 (59.6)	
Alcohol abuse			0.690			0.041
Yes	49 (17.2)	4 (14.3)		179 (80.3)	80 (89.9)	
No	235 (82.8)	24 (85.7)		44 (19.7)	9 (10.1)	
Smoking			0.487			0.634
Yes	74 (26.1)	9 (32.1)		61 (27.3)	22 (24.7)	
No	210 (73.9)	19 (67.9)		162 (72.7)	67 (75.3)	
Smoking index			0.829			0.736
≤400	239 (84.2)	24 (85.7)		187 (83.9)	76 (85.4)	
>400	45 (15.8)	4 (14.3)		36 (16.1)	13 (14.6)	
Family history of malignant tumor			0.446			0.406
Yes	45 (15.8)	6 (21.4)		34 (15.2)	17 (19.1)	
No	239 (84.2)	22 (78.6)		189 (84.8)	72 (80.9)	
Comorbidities			0.408			0.623
Yes	119 (41.9)	14 (50.0)		97 (43.5)	36 (40.4)	
No	165 (58.1)	14 (50.0)		126 (56.5)	53 (59.6)	
Treatment modality			0.560			0.675
Surgery alone	198 (69.7)	21 (75.0)		155 (69.5)	64 (71.9)	
Surgery with adjuvant therapy	86 (30.3)	7 (25.0)		68 (30.5)	25 (28.1)	
Surgical approach			0.445			0.001
VATS	250 (88.0)	26 (92.9)		189 (84.8)	87 (97.8)	
Open	34 (12.0)	2 (7.1)		34 (15.2)	2 (2.5)	
Laterality			0.408			0.485
Left	139 (48.9)	16 (57.1)		108 (48.4)	47 (52.8)	
Right	145 (51.1)	12 (42.9)		115 (51.6)	42 (47.2)	
Lobe			0.942			20.941
Upper	69 (24.3)	7 (25.0)		56 (25.1)	20 (22.5)	
Middle	15 (5.3)	1 (3.6)		12 (5.4)	4 (4.5)	
Lower	194 (68.3)	19 (67.8)		150 (68.3)	63 (70.8)	
Overlapping	6 (2.1)	1 (3.6)		5 (2.2)	2 (2.2)	
Tumor Size, cm			0.344			0.069
≤3	178 (62.7)	15 (53.6)		145 (65.0)	48 (53.9)	
>3	106 (37.3)	13 (46.4)		78 (35.0)	41 (46.1)	
Genetic test			0.481			<0.001
NA	107 (37.7)	9 (32.1)		95 (42.6)	21 (23.6)	
None	77 (27.1)	5 (17.9)		66 (29.6)	16 (18.0)	
KRAS mutation	74 (26.0)	11 (39.3)		46 (20.6)	39 (43.8)	
EGFR mutation	11 (3.9)	2 (7.1)		8 (3.6)	5 (5.6)	
Others	15 (5.3)	1 (3.6)		8 (3.66)	8 (9.0)	0.623
T stage			0.422			0.201
T1‐2	237 (83.5)	25 (59.3)		191 (85.7)	71 (87.179.8)	
T3‐4	47 (16.5)	3 (10.7)		32 (14.3)	18 (12.920.2)	
N stage			0.012			0.184
N0	257 (90.5)	21 (75.0)		202 (90.6)	76 (90.385.4)	
N1‐2	27 (9.5)	7 (25.0)		21 (9.4)	13 (9.714.6)	
TNM stage			0.004			0.029
I/II	243 (85.6)	18 (64.3)		193 (86.6)	68 (80.676.4)	
III	41 (14.4)	10 (35.7)		30 (13.4)	21 (19.423.6)	
CD8^+^ TIL density, mm^2^						
Median (IQR)	313.2 (187.1–498.8)	398.0 (276.2–670.4)	0.054^*^	288.0 (170.6–467.4)	436.5 (26.4–689.7)	<0.001^*^
Mean ± SD	394.1 ± 306.1	478.9 ± 283.4	0.160^**^	360.4 ± 277.2	505.3 ± 344.9	<0.001^**^
Positive (≥148.7 mm^2^), *n* (%)	231 (81.3)	25 (89.3)	0.296^***^	176 (78.9)	80 (89.9)	0.023^***^
CD8+ TIL percentage, %						
Median (IQR)	8.4 (5.0–12.5)	7.4 (6.2–13.2)	0.621^*^	8.0 (4.9–11.5)	9.7 (5.7–13.8)	0.016^*^
Mean ± SD	9.5 ± 6.2	10.0 ± 6.0	0.656^**^	9.0 ± 5.7	11.1 ± 7.1	0.005^**^
Positive (≥3. 8), n (%)	243 (85.6)	25 (89.3)	0.589^***^	187 (83.9)	81 (91.0)	0.101^***^
Overall survival, *n* (%)			0.715^****^			0.112^****^
Yes	258 (90.9)	26 (92.9)		198 (88.8)	86 (96.6)	
No	26 (9.1)	2 (7.1)		25 (11.2)	3 (3.4)	
Relapse‐free survival, *n* (%)			0.535^****^			0.861^****^
Yes	237 (83.5)	25 (89.3)		184 (82.5)	78 (87.6)	
No	47 (16.5)	3 (10.7)		39 (17.5)	11 (12.4)	

Abbreviations: CPS, combined positive score; IQR, interquartile range; NA, not applicable; SD, standard deviation; TNM, tumor node metastasis stage; TPS, tumor positive score; VATS, video‐assisted thoracoscopic surgery.

^*^Mann–Whitney rank‐sum test.

^**^Student *t*‐test.

^***^Chi‐squared test.

^****^Log‐rank test.

### 2.4 Association Between CD8^+^ TILs and PD‐L1 Expression

Significant association was observed between PD‐L1 expression and the level of CD8^+^ TIL infiltration in IMA patients (Tables 1 and 2). Therefore, the potential association between PD‐L1 expression and CD8^+^ TIL infiltration level was further examined by comparing the differences in the percentage and density of CD8^+^ TILs between PD‐L1‐negative and PD‐L1‐positive patients. Patients who were PD‐L1‐positive had significantly greater percentages of CD8^+^ TILs (TPS criteria: 10.0% vs. 9.5%, *p* = 0.621; Figure 3A; CPS criteria: 11.1% vs. 9.0%, 0.016; Figure 3B) and greater density of CD8^+^ TILs (TPS criteria: 478.9 vs. 394.1 cells/mm^2^, *p* = 0.054; Figure 3C; CPS criteria: 505.3 vs. 360.4 cells/mm^2^, *p* < 0.001; Figure 3D) than PD‐L1‐negative patients.

To better assess the association between the CD8^+^ TIL infiltration level and PD‐L1 expression, patients were stratified into low‐ (CD8^+^ TIL percentage: <6.04%; CD8^+^ TIL density: <232.6 cells/mm^2^), medium‐ (CD8^+^ TIL percentage: 6.04%–10.58%; CD8^+^ TIL density: 232.6–448.4 cells/mm^2^), and high‐ (CD8^+^ TIL percentage: >10.58%; CD8^+^ TIL density: >448.4 cells/mm^2^) infiltration groups according to the tertiles. Then, the proportions of PD‐L1‐positive patients among the three CD8^+^ TIL infiltration level groups were compared. As expected, increased CD8^+^ TIL percentage (Figure 3E) and density (Figure 3F) were found to be correlated with increased PD‐L1 expression, as evaluated by CPS (*p* for trend = 0.006 and *p* for trend <0.001, respectively), whereas no significant correlation was observed when evaluated by TPS (*p* for trend = 0.660 and *p* for trend = 0.184, respectively). Specifically, 24.04% and 20.19% of patients were PD‐L1‐positive (CPS ≥1) in the low CD8^+^ TIL percentage and density group, respectively, whereas in the high CD8^+^ TIL percentage and density group, 40.38% and 40.38% of patients were PD‐L1‐positive, respectively. Furthermore, we plotted a Sankey diagram to illustrate the overall distribution of PD‐L1 expression versus CD8^+^ T‐cell infiltration (Figure 3G).

### 2.5 Prognostic Implication of PD‐L1 Expression and CD8^+^ TIL Infiltration

We conducted evaluations to ascertain the connections between PD‐L1 expression or CD8^+^ TIL infiltration and long‐term survival. Univariate Cox regression and Kaplan‒Meier curve analyses indicated that PD‐L1 expression was not significantly associated with RFS (TPS: hazard ratio [HR] = 0.69, 95% confidence interval [CI]: 0.21–2.22, *p* = 0.535; CPS: HR = 0.94, 95% CI: 0.48–1.85, *p* = 0.861) or OS (TPS: HR = 0.76, 95% CI: 0.18–3.22, *p* = 0.715; CPS: HR = 0.38, 95% CI: 0.11–1.26, *p* = 0.112) (Figure S1A‒D).

On the other hand, based on the optimal cutoff values for the percentage of CD8^+^ TILs and density for predicting RFS, patients were categorized into different groups. Patients exhibiting a high percentage of CD8+ TILs (≥3.8%) demonstrated significantly improved RFS (HR = 0.33, 95% CI: 0.18–0.60, *p* < 0.001), whereas there was no notable difference in OS (HR = 0.57, 95% CI: 0.23–1.42, *p* = 0.230) between the two groups. Similarly, patients exhibiting high CD8^+^ TIL density (≥148.7 cells/mm^2^) had markedly longer RFS (HR = 0.42, 95% CI: 0.23–0.75, *p* = 0.004) and longer OS (HR = 0.38, 95% CI: 0.18–0.83, *p* = 0.016) (Figure S1E‒H).

### 2.6 Subgroup Analysis Based on Adjuvant Therapy Status

Considering the potential confounding effect of adjuvant therapy, we evaluated the prognostic value of CD8^+^ TILs and PD‐L1 within subgroups stratified by treatment. For RFS, a higher CD8^+^ TIL percentage was a strong predictor of improved outcome in patients who underwent surgery alone (HR = 0.20, 95% CI: 0.07–0.54, *p* = 0.002) but not in those who received adjuvant therapy (HR = 0.73, 95% CI: 0.35–1.55, *p* = 0.412). Similarly, higher CD8^+^ TIL density was significantly associated with longer RFS in the surgery‐only subgroup (HR = 0.31, 95% CI: 0.12–0.85, *p* = 0.023) but not in the adjuvant therapy subgroup (HR = 0.72, 95% CI: 0.35–1.50, *p* = 0.387) (Figure S2A). For OS, higher CD8^+^ TIL density showed a nonsignificant trend toward benefit in the surgery‐only subgroup (HR = 0.37, 95% CI: 0.13–1.07, *p* = 0.067). In the adjuvant therapy subgroup, neither CD8^+^ TIL percentage (HR = 0.88, 95% CI: 0.24–3.28, *p* = 0.848) nor density (HR = 0.47, 95% CI: 0.15–1.49, *p* = 0.201) was significantly associated with OS (Figure S2B). PD‐L1 expression, evaluated by either TPS or CPS, was not significantly associated with RFS or OS in the overall cohort or in either treatment subgroup (Figure S2A,B).

### 2.7 Construction of a Four‐Category Immune Phenotype Classification

**FIGURE 4:**
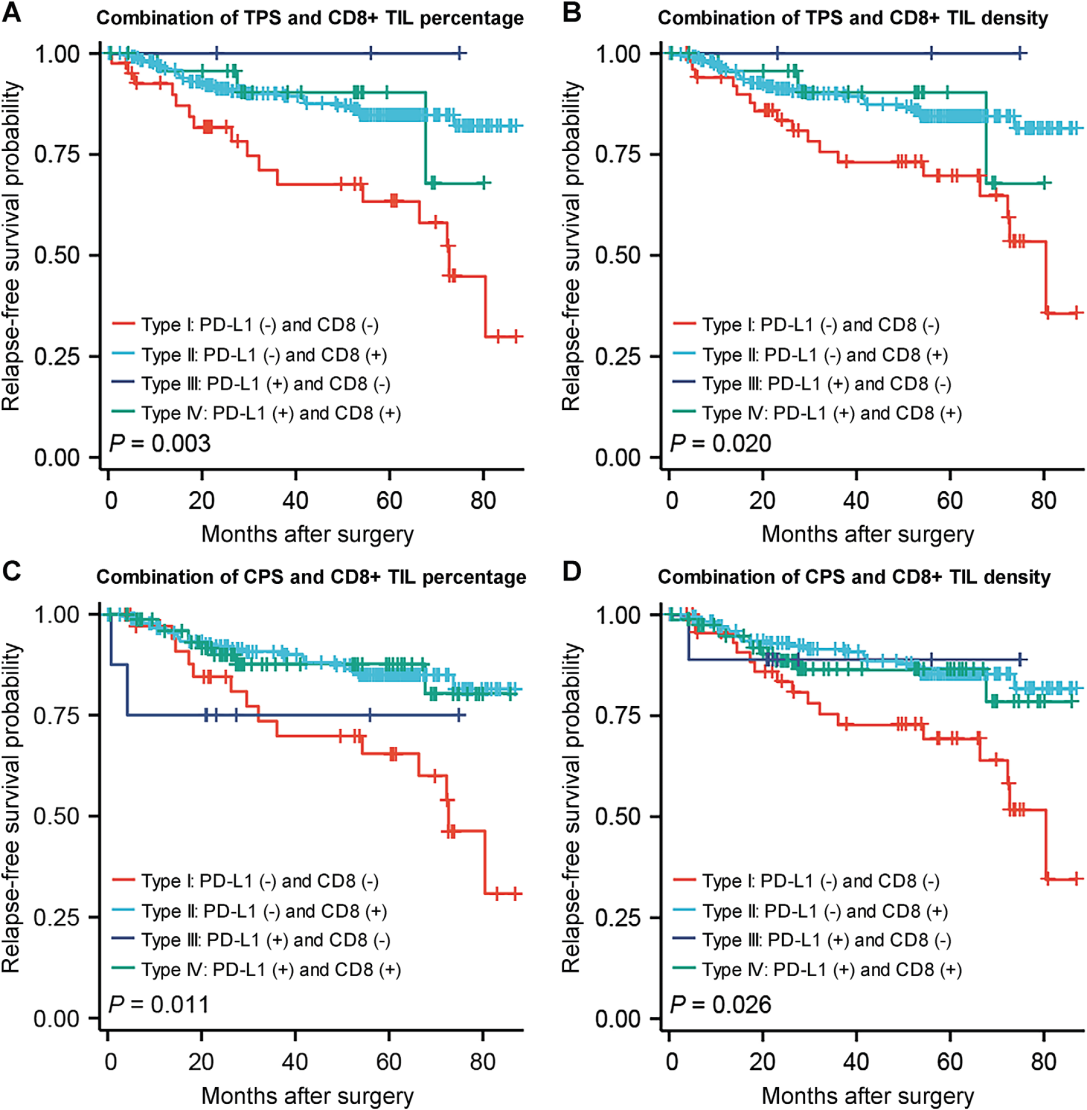
The prognostic value of different combinations of PD‐L1 expression evaluation methods and CD8^+^ T‐cell infiltration level metrics in predicting relapse‐free survival (RFS). (A) TPS criteria and CD8^+^ TIL percentage; (B) TPS criteria and CD8^+^ TIL density; (C) CPS criteria and CD8^+^ TIL percentage; (D) CPS criteria and CD8^+^ TIL density.

**FIGURE 5:**
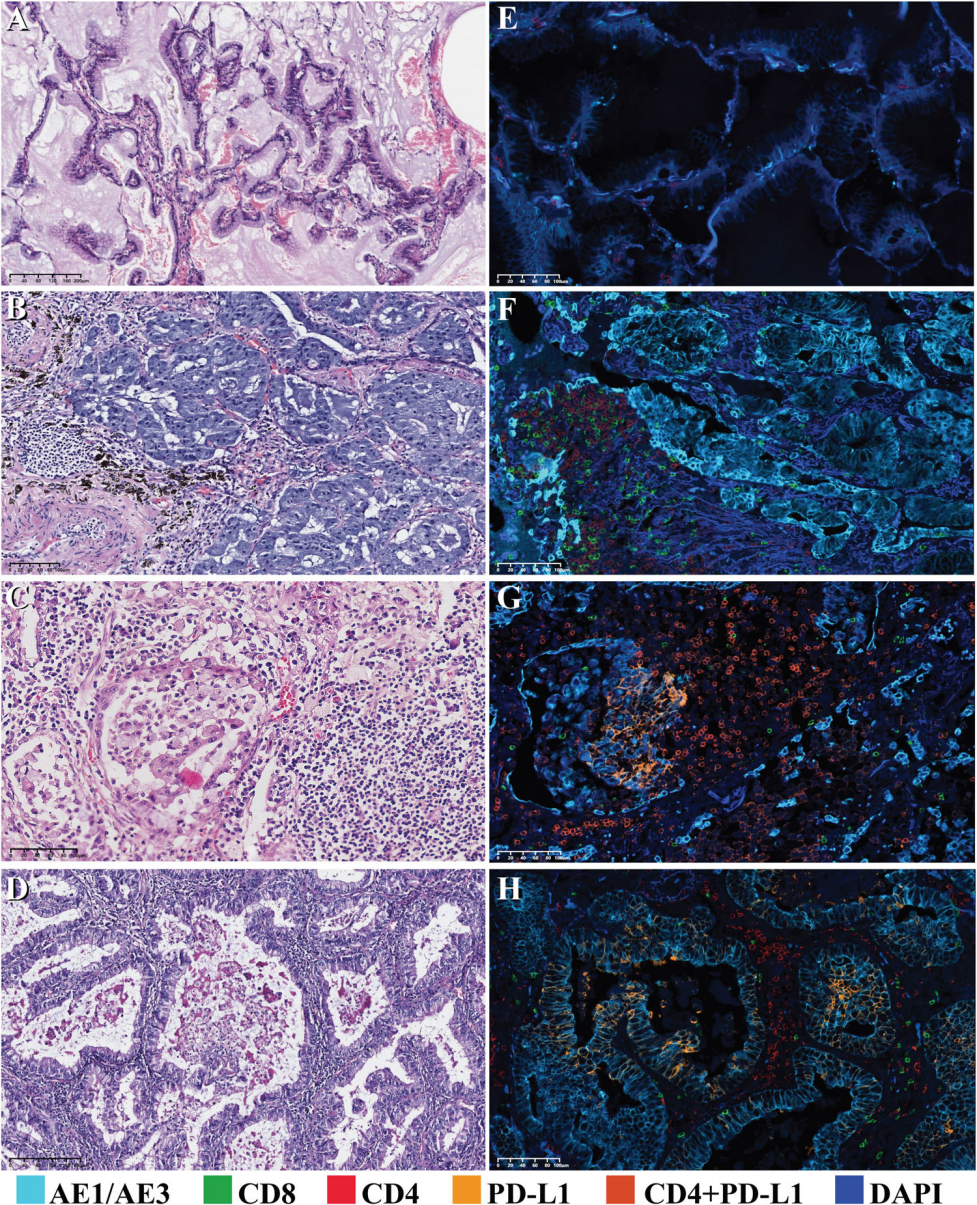
Representative IHC and mIF images of samples from patients with the four different immune phenotypes. (A–D) The histomorphology (HE staining, ×200) corresponding to the four immune phenotypes; (E–H) The results of multiple immunofluorescence staining (×200) of the four immune phenotypes; (E) shows that there were almost no CD8‐positive lymphocytes in the tumor, and there was no expression of PD‐L1 (type I); (F) shows that there were more CD8‐positive lymphocytes in the tumor stroma, but there was no expression of PD‐L1 in tumor cells and lymphocytes (type II); (G) shows moderate expression of PD‐L1 in CD4‐positive lymphocytes and tumor cells, but almost no CD8‐positive lymphocytes were present (type III); and (H) shows a greater number of CD8‐positive lymphocytes, along with high expression of PD‐L1 in tumor cells (type IV).

Treatment strategies for cancer have been proposed, incorporating considerations of PD‐L1 expression and TIL infiltration, particularly emphasizing CD8^+^ TILs. We further grouped patients according to PD‐L1 expression and CD8^+^ TIL infiltration. Given the two evaluation criteria for both PD‐L1 expression (TPS and CPS) and the degree of CD8^+^ TIL infiltration (percentage and density) in this study, to determine which combination could best distinguish patients with markedly different prognoses, we compared the value of four different combinations in predicting patient RFS (Figure 4A‒D). The grouping details and patient numbers for each combination are shown in Table S2. The results demonstrated that the TPS combined with the percentage of CD8^+^ TILs could best distinguish patients; patients with the four different immunophenotypes based on this model had significant differences in RFS (log‐rank *p* = 0.003). Specifically, patients who were PD‐L1‐negative (according to the TPS criterion) and had a low percentage of CD8^+^ T‐cells (Type I) had markedly worse RFS than patients in other groups (Figure 4A). Thus, a four‐category immune phenotype classification was constructed based on the combination of PD‐L1 TPS with CD8^+^ TIL percentage. Representative IHC (Figure 5A‒D) and mIF (Figure 5E‒H) images of samples from patients with the four different immune phenotypes are displayed, which provides a visual representation of the immune microenvironment phenotypes.

### 2.8 Multivariate Analysis Identified Independent Prognostic Biomarkers

Next, we investigated whether the PD‐L1 expression and CD8^+^ TIL infiltration were significant independent predictors of prognosis in IMA patients. Univariate analyses revealed that RFS was significant with factors including gender, alcohol consumption, smoking status, treatment modality, tumor site, tumor size, T stage, N stage, TNM stage, as well as the percentage and density of CD8^+^ TILs. OS was significantly correlated with factors including gender, alcohol consumption, smoking status, surgical approach, tumor size, T stage, N stage, TNM stage, and CD8^+^ TIL density (Table S3). Considering the clinical implications, univariate Cox regression results, and collinearity of the final model, we included TPS score, the percentage of CD8^+^ TILs, sex, age, smoking status, surgical approach, tumor size, TNM stage, and treatment modality in the final multivariate model (Table 3). The findings indicated that the percentage of CD8^+^ TILs independently served as an independent prognostic factor for patient RFS (adjusted HR = 0.35, 95% CI: 0.12–0.69, *p* = 0.002) but not OS (adjusted HR = 0.67, 95% CI: 0.25–1.78, *p* = 0.422). PD‐L1 positivity (TPS ≥1%) was not significantly associated with improved RFS (adjusted HR = 0.42, 95% CI: 0.12–1.46, *p* = 0.174) or OS (adjusted HR = 0.40, 95% CI: 0.09–1.85, *p* = 0.239), though the HRs suggested a potential protective direction. TNM stage (III vs. I) was a robust independent predictor of both RFS (adjusted HR = 4.71, 95% CI: 2.15–10.33, *p* < 0.001) and OS (adjusted HR = 3.50, 95% CI: 1.16–10.59, *p* = 0.027) in IMA patients. Notably, the receipt of adjuvant therapy was associated with worse RFS (adjusted HR = 2.59, 95% CI: 1.39–4.84, *p* = 0.003) compared with surgery alone, though no significant association with OS (adjusted HR = 0.86, 95% CI: 0.38–1.93, *p* = 0.710) was observed.

**TABLE 3:** Multivariate Cox regression analysis of PD‐L1 expression, CD8 infiltration, and clinicopathological characteristics in IMA patients.

		Relapse‐free survival		Overall survival	
Variable		HR (95% CI)	*p*‐value	HR (95% CI)	*p*‐value
PD‐L1 expression by TPS	Positive vs. Negative	0.42 (0.12–1.46)	0.174	0.40 (0.09–1.85)	0.239
CD8^+^ TIL percentage, %	Positive vs. Negative	0.35 (0.18–0.69)	0.002	0.67 (0.25–1.78)	0.422
Gender	Male vs. Female	2.11 (0.96–4.63)	0.062	1.67 (0.52–5.38)	0.391
Age	>60 vs. ≤60	1.41 (0.76–2.64)	0.274	1.50 (0.67–3.38)	0.322
Smoking	Yes vs. No	1.12 (0.52–2.41)	0.779	2.03 (0.68–6.11)	0.207
Surgical approach	VATS vs. Open	1.34 (0.62–2.93)	0.459	0.65 (0.26–1.58)	0.340
Tumor size, cm	>3 vs. ≤3	1.77 (0.85–3.68)	0.125	2.07 (0.71–5.98)	0.180
TNM stage	II vs. I	1.88 (0.77–4.59)	0.167	1.56 (0.47–5.16)	0.464
	III vs. I	4.71 (2.15–10.33)	<0.001	3.50 (1.16–10.59)	0.027
Treatment modality	Surgery with adjuvant therapy vs. Surgery alone	2.59 (1.39–4.84)	0.003	0.86 (0.38–1.93)	0.710

Abbreviations: CI, confidence interval; CPS, combined positive score. (–); HR, hazard ratio; TNM, tumor node metastasis stage; VATS, video‐assisted thoracoscopic surgery.

### 2.9 Tumor Immune Microenvironment Phenotypes and Response to Immunotherapy

To investigate the association between the defined four immune phenotypes and response to ICIs, we established an expanded cohort of IMA patients who received ICI therapy (*n* = 20). Within this cohort, assessment of PD‐L1 (TPS) and CD8^+^ TIL percentage classified patients into the four defined immune phenotypes: Type I (*n* = 4), Type II (*n* = 11), Type III (*n* = 1), and Type IV (*n* = 4). The treatment timeline, regimen, number of ICI cycles, best observed response, and outcome for each patient were detailed in Figure S3, which also summarizes the distribution of responses within each immune phenotype. No patient achieved a complete response (CR). The distribution of best overall responses differed notably among the immune phenotypes. Among Type I patients, stable disease (SD) was observed in three cases and progressive disease (PD) in 1 case. Type II patients showed 1 partial response (PR), 6 SD, and 4 PD. The sole Type III patient achieved SD. Notably, Type IV was associated with the most favorable outcomes, with three patients achieving PR and one patient achieving SD, reflecting the highest objective response rate. These observations suggest that the Type IV (PD‐L1^+^/CD8^+^) immune phenotype may be associated with a greater likelihood of clinical benefit from ICI therapy in IMA.

### 2.10 Single‐Cell Profiling of the Tumor Immune Microenvironment in IMA

Integrated analysis of single‐cell RNA sequencing data from 2 IMA and 6 nonmucinous adenocarcinoma (NMA) samples was performed to transcriptionally profile the tumor microenvironment. Following standard preprocessing, cells were categorized into eight major lineages: T and NK cells, B cells, myeloid cells, mast cells, fibroblasts, endothelial cells, epithelial cells, and proliferating cells (Figure S4A–D). Further subclustering of the T and NK cell compartments identified ten distinct subsets, including Treg_CD4_FOXP3, Tex_CD4_PDCD1, Naive_CD4_CCR7, Tmem_CD4_ANXA1, Teff_CD4_FOSB, Tfh_CD4_CXCR5, Teff_CD8_GZMK, Teff_CD8_ZNF683, NK_CD56bright, and NK_CD56dim (Figure S4E,G). Comparative analysis revealed significant differences in T cell composition between IMA and NMA. Notably, the proportion of a defined effector CD8^+^ T cell subset (Teff_CD8_ZNF683) was substantially lower in IMA (4.4%) than in NMA (25.3%) (Figure S4F). Furthermore, expression of the exhaustion marker PD‐1 within the effector T cell compartment was significantly reduced in IMA (*p* = 2.57e‐13) (Figure S4H). These transcriptional findings align with our protein‐level observations and provide mechanistic insight into the immune microenvironment in IMA.

## 3 Discussion

The rapid development of ICIs, particularly anti‐PD‐L1 and anti‐PD‐1 antibodies, has led to the development of new therapeutic strategies for an increasing number of patients with advanced NSCLC, even those with negative PD‐L1 expression [12, 13]. However, not all patients can benefit from these treatments. Therefore, understanding the tumor microenvironment is crucial for identifying patients who can benefit from ICI therapy. As a distinct and rare histological subtype of NSCLC, IMA displays more aggressive local invasion, higher rates of multifocal and metastatic disease, and a poorer prognosis. Unfortunately, limited data exist regarding the tumor immune microenvironment of this rare NSCLC variant and its relevance to clinical outcomes, as well as the efficacy of immunotherapy.

With this large multicenter study of 312 IMA patients who underwent surgical resection, we provide novel insights into the landscape of PD‐L1 expression and CD8^+^ TIL infiltration and the prognostic significance of these features. A key finding was that the proportion of PD‐L1‐positive IMA patients (9.0% according to the TPS criteria; 28.5% according to the CPS criteria) was markedly lower in this study than in previously reports (46% for TPS ≥1% and 60% for CPS ≥1) on unselected NSCLC patients [4, 14]. The reasons underlying this discrepancy are unclear, but they could be linked to the unique biological characteristics of IMA. Abundant extracellular mucin might function as a physical obstacle, restricting immune cell infiltration into the tumor microenvironment and potentially hindering PD‐L1 induction [15]. Mutational profiles also differ between IMA and conventional lung adenocarcinomas; IMA has a lower overall number of mutations, so PD‐L1 may not be upregulated to the same degree [16]. Indeed, we did not observe PD‐L1 expression to independently influence prognosis in IMA, similar to the findings of a previous study [11]. Our results indicate that PD‐L1 expression has limited utility as a prognostic biomarker in IMA patients and that distinct prognostic models incorporating alternative immune parameters are needed.

A key novel finding was the positive correlation between PD‐L1 expression and CD8+ TIL percentage and density. Patients with PD‐L1‐positive tumors demonstrated significantly greater CD8+ TIL densities and percentages than PD‐L1‐negative patients. This correlation between PD‐L1 and TILs has been reported across diverse solid tumor types, including melanoma, colorectal cancer, breast cancer, and renal cell carcinoma [17, 18]. The concomitant presence of PD‐L1 and CD8^+^ TILs is understood to reflect adaptive immune resistance, whereby PD‐L1 is upregulated on tumor cells in response to immunostimulatory cytokines such as interferon‐gamma produced by activated T cells [19]. Our study reinforces the interdependence of the PD‐1/PD‐L1 axis and CD8^+^ T‐cell activity specifically within the IMA microenvironment. This finding has implications in identifying patients most suitable for PD‐1/PD‐L1 blockade, as our approach to subgrouping patients according to both the percentage of TILs and PD‐L1 expression may increase the clinical benefit of immunotherapy.

This study further revealed that the immune microenvironment subtypes of IMA are indeed associated with the efficacy of immunotherapy. The observation that patients with the Type IV (PD‐L1^+^/CD8^+^) phenotype exhibited the highest objective response rate aligns with the biological concept of adaptive immune resistance. This phenotype likely represents a tumor microenvironment in which pre‐existing antitumor immunity, as evidenced by CD8^+^ T‐cell infiltration, is actively suppressed by the PD‐1/PD‐L1 axis, making it particularly susceptible to checkpoint blockade. Conversely, the limited benefit observed in Type I (PD‐L1^−^/CD8^−^) patients underscores the challenge of treating immunologically “cold” or noninflamed tumors with single‐agent ICI therapy. While these results are encouraging and suggest our model may help identify IMA patients more likely to benefit from immunotherapy, we acknowledge the limitations of this exploratory analysis, including its small sample size and retrospective nature. The limited efficacy in the cohort also highlights that IMA remains a challenging subtype for immunotherapy. This intrinsic resistance may be partly attributed to the distinct molecular profile of IMA, which is frequently driven by Kirsten rat sarcoma viral oncogene homolog (KRAS) mutations, particularly the G12D variants [20]. Liu et al. found that the KRAS‐G12D mutation is associated with a more immunosuppressive tumor microenvironment and primary resistance to anti‐PD‐1/PD‐L1 therapy in NSCLC patients [21]. Notably, subgroup analysis revealed that the prognostic strength of CD8^+^ T‐cell infiltration as an independent biomarker was markedly attenuated in patients who received adjuvant therapy, highlighting a significant clinical–biological interaction. The lymphodepletion induced by adjuvant chemotherapy itself may obscure the original prognostic signal of the intratumoral CD8^+^ T‐cell infiltration [22]. More importantly, this finding suggests that the benefit derived from adjuvant therapy may not be uniform across different immune phenotypes. Our data indicated that the already deficient effector CD8^+^ T‐cells in IMA may be further compromised following adjuvant chemotherapy, which could explain the diminished prognostic value of CD8^+^ TILs in this subgroup.

When assessing prognosis, higher CD8^+^ TIL densities and percentages were identified as independent favorable prognostic biomarkers for RFS in IMA patients. CD8^+^ cytotoxic T‐cells can directly target and lyse tumor cells by releasing perforin, granzyme, and other effector molecules [23]. Their accumulation in the tumor microenvironment provides evidence of ongoing antitumor immune activity. Our results align with those of previous studies across diverse epithelial malignancies, demonstrating that CD8^+^ TIL density is a robust prognostic biomarker superior to PD‐L1 expression [24, 25]. Integration of quantitative CD8^+^ TIL assessment could substantially improve prognostic models and immunotherapeutic strategies for IMA. In this study, patients characterized by negative PD‐L1 expression and low CD8+ TIL infiltration demonstrated an immunologically “cold” phenotype, which was associated with significantly worse RFS. The development of immune‐based classifications could improve the risk stratification of IMA patients with “cold” tumors by distinguishing those requiring intensified treatment regimens from those suitable for ICI therapy.

Further analysis of CD8^+^ T cells suggests that IMA is characterized by a fundamental deficit in both the quantity and functional state of T cells. Compared with NMA, the proportion of a defined effector CD8^+^ T cell subset (Teff_CD8_ZNF683) was significantly lower within the IMA tumor microenvironment, and these effector T cells expressed lower levels of PD‐1. The absence of effective effector T cell clones is likely a root cause for the failure to mount an efficient antitumor immune response. In NSCLC, the functional state of CD8^+^ T cells located at the tumor invasive margin has been linked to lymph node metastasis and recurrence‐free survival [26, 27]. The paucity of functional effector T cells in IMA may partially account for its generally poorer prognosis and lower response rates to anti‐PD‐1/PD‐L1 therapy. This indicates that future immunotherapeutic strategies for IMA may need to look beyond merely mobilizing the existing T‐cell repertoire and instead focus on reshaping or activating a new and fully functional effector T‐cell pool.

This work provides the first systematic delineation of the unique immune microenvironment in a large, rigorously defined cohort of pure IMA, establishing a novel immune phenotype framework with potential clinical utility. We focused on a clearly defined but understudied rare subtype. The multi‐center, large‐sample design ensures the reliability of the conclusions. A novel, clinically relevant immune classification framework was established, with preliminary validation in an immunotherapy cohort. Furthermore, a combination of histomorphology, prognostic analysis, and mechanism‐explorative bioinformatics based on single‐cell transcriptomic data added depth to the findings. Collectively, these efforts address a significant knowledge gap and offer a new paradigm for understanding and managing this distinct form of lung adenocarcinoma.

Several limitations should be acknowledged. The retrospective design and single‐country study population present inherent selection biases that may affect the generalizability of our findings. Although we have explored the value of the immune phenotyping framework in the immunotherapy cohort, the patterns of PD‐L1 expression and CD8^+^ TIL infiltration reported here may not fully represent the global IMA population, particularly patients with advanced, nonresectable disease. While we identified quantitative and compositional differences in CD8^+^ T cells via single‐cell profiling, we did not perform functional analyses of TILs, including studies on cytokine production and cytotoxicity. The evaluation of additional emerging immune biomarkers in lung adenocarcinoma, such as tumor mutation burden, lymphocyte‐activating gene 3 (LAG‐3), and T‐cell immunoglobulin and mucin‐domain containing molecule 3 (TIM‐3), was not conducted. Changes in the immune microenvironment over time or following systemic therapy were not assessed. Prospective external validation is warranted to confirm the findings. The combination of multiplex immunohistochemistry, cytometric analysis, and transcriptomic immune profiling could provide further insights into the complex immune landscape of IMA. Longitudinal biopsies evaluating dynamic changes in PD‐L1 and CD8^+^ TILs and other parameters before and after treatment are also needed. Nevertheless, our study provides a strong rationale for further developing CD8^+^ TILs as biomarkers to guide the management of IMA in the era of cancer immunotherapy.

In summary, this large multicenter investigation of IMA surgical specimens provides novel insights into the prognostic relevance of PD‐L1 expression and CD8^+^ TIL infiltration. We determined that PD‐L1 positivity rates are markedly lower in IMA than in conventional NSCLC. In contrast, the percentage and density of CD8^+^ TILs were identified as independent favorable prognostic factors and immunotherapeutic biomarkers warranting further clinical investigation in this rare lung cancer subtype. Our findings support the integration of PD‐L1 expression and CD8^+^ TIL assessment to improve the risk stratification of patients, guide the selection of optimal candidates for immunotherapy, and ultimately improve the poor outcomes of IMA patients.

## 4 Materials and Methods

### 4.1 Study Patients

The present multicenter retrospective study involved the collection and analysis of surgical pathology specimens from 312 IMA patients. All included patients underwent curative surgical resection between January 2016 and December 2024 at three tertiary hospitals/cancer treatment research centers in China. The detailed distribution of patients is listed in Table S4. Pathological TNM staging was assessed according to the American Joint Committee on Cancer Staging System (the eighth edition). Patients meeting the following criteria were eligible for inclusion: (1) age ≥18 years, (2) pathological diagnosis of pure IMA (mucinous component ≥90%), and (3) underwent curative surgical resection. Patients were excluded if they had received preoperative neoadjuvant therapy or were lost to follow‐up. This study was approved by the ethics committees of the National Cancer Center (ethics number: 22/244‐3446), with the written informed consent from patients waived.

### 4.2 Pathological Diagnosis of IMA

The pathological diagnosis of IMA was established in strict accordance with the 2021 World Health Organization (WHO) standards and guidelines of Thoracic Tumors (fifth edition) [1]. Diagnosis was primarily based on characteristic histomorphological features, specifically the presence of tumor cells with goblet or columnar morphology containing abundant intracytoplasmic mucin. To support the diagnosis and critically exclude metastatic mucinous adenocarcinoma, immunohistochemical staining for markers including TTF‐1, HNF4α, and CDX2 was performed when necessary. All hematoxylin and eosin‐stained slides underwent independent evaluation by two expert pathologists (Jia Jia and Liyan Xue), and any discrepancies were resolved by discussion. Only patients who underwent complete (R0) surgical resection, confirmed by pathological assessment of margins, were included in the final cohort; cases with microscopic (R1) or macroscopic (R2) residual disease were excluded. It is important to clarify that the term “invasive” herein refers specifically to the histopathological growth pattern as defined by the WHO classification (i.e., tumor cells infiltrating the stroma), which is distinct from the clinical TNM staging of the tumor.

### 4.3 Genetic Test

To characterize the driver mutation landscape of IMA, we retrospectively collected the results of postoperative genetic testing for all included patients. Targeted polymerase chain reaction (PCR)‐based assays and next‐generation sequencing (NGS) were used in clinical practice. The targeted PCR assays primarily covered core driver genes such as epidermal growth factor receptor (EGFR), KRAS, and anaplastic lymphoma kinase (ALK). NGS typically employs panels covering dozens to hundreds of cancer‐related genes, enabling simultaneous detection of single‐nucleotide variants, insertions/deletions, copy number variations, and gene fusions. Based on the test reports, patients were categorized into four statuses, including not applicable (NA), none driver mutation detected (None), EGFR mutation, KRAS mutation, and other driver mutations (Others). The “Others” category consolidates all definitively identified driver gene alterations other than EGFR and KRAS, including but not limited to rearrangements in ALK, ROS1, and RET, as well as mutations or alterations in MET, ERBB2, BRAF V600E, and NRG1 fusions.

### 4.4 Study Endpoints

The primary endpoints were RFS and OS. RFS was delineated as the period (unit: months) from lung resection to relapse, metastasis, or the final follow‐up assessment. On the other hand, OS was characterized as the period (unit: months) from lung resection to death or the final follow‐up assessment.

### 4.5 Immunohistochemistry

For IHC analysis, 4 µm‐thick formalin‐fixed and paraffin‐embedded (FFPE) sections from large resected specimens were prepared on glass slides. The slides were deparaffinized and rehydrated through a series of ethanol dilutions. Endogenous peroxidase activity was then blocked by incubating the slides in 3% H_2_O_2_ solution for 15 min. Antigen retrieval was subsequently performed by microwaving the sections in EDTA buffer (pH 9.0) for 30 min. Next, 10% normal serum was used to block tissue samples before overnight incubation at 4°C with an antibody against PD‐L1 to stain for PD‐L1 expression. For CD8 staining, the sections were incubated overnight at 4°C with an antibody against CD8. This was followed by a 30 min room temperature incubation with a goat anti‐mouse/rabbit secondary antibody. Finally, 3,3’‐diaminobenzidine was used to visualize antibody binding, and hematoxylin counterstaining was performed. The comprehensive details regarding the reagents utilized are provided in Table S5. All quantitative measures of PD‐L1 expression and CD8^+^ TILs, which were used for all subsequent correlative, survival, and immune phenotyping analyses, were obtained solely from IHC.

### 4.6 mIF Staining

FFPE samples underwent staining utilizing the BOND‐MAX staining platform with Leica Microsystems (Buffalo Grove, IL, USA). The staining process employed a 6‐color kit from Abcarta in Suzhou, China. The markers used for staining included PD‐L1, CD4, CD8, and cytokeratin‐pan. To establish positive and negative controls, FFPE tissues from normal human tonsil were stained. All stained slides were then digitally scanned by the KF‐PRO‐005 scanner (×40, KFBIO, Ningbo, China), followed by manual review to ensure consistency and accuracy. In contrast to the quantitatively scored IHC, mIF was employed solely for qualitative, illustrative purposes. Its primary aim was to visualize the spatial distribution and co‐localization of PD‐L1 expression and CD8^+^ T cells within the tumor microenvironment, particularly within the four defined immune phenotypes.

### 4.7 Evaluation of PD‐L1 Expression

Two expert pathologists, unaware of patient information and outcomes, assessed the PD‐L1 IHC staining and distinguished tumor cells from immune cells based on combined morphological evaluation of corresponding H&E and IHC‐stained sections. Any cases with discrepant results were jointly reviewed under a multiheaded microscope and discussed until a consensus was reached. All sections contained a minimum of 100 tumor cells with areas of necrosis excluded. For TPS evaluation, only tumor cells exhibiting partial or complete linear membranous staining were considered positive, while cytoplasmic staining was disregarded. The TPS score was defined as the percentage of viable tumor cells with membranous PD‐L1 staining. Similarly, tumor‐infiltrating immune cells, including TILs and tumor‐associated macrophages (TAMs), were also evaluated and counted if membranous or cytoplasmic staining of the respective markers was detected. The peritumoral area was delineated as 500 µm from the tumor border and nests into the surrounding tissue. The CPS was derived by dividing the total count of PD‐L1‐positive cells (tumor cells, TILs, and TAMs) by the number of viable tumor cells, and then multiplying the result by 100. In the present study, both a TPS ≥1% and a CPS ≥1 were regarded as indicators of PD‐L1‐positive.

### 4.8 Quantification of the CD8^+^ TIL Infiltration Level

To quantify the intratumoral and peritumoral infiltration of CD8^+^ TILs, digital image analysis was performed on whole‐slide sections stained with an anti‐CD8 antibody. The slides were initially scanned at high resolution (400×) using a Panoramic MIDI II slide scanner (3DHISTECH Ltd, Hungary). Subsequently, a trained pathologist delineated the tumor areas on the digital slides using CaseViewer software (version 2.4.0, 3DHISTECH Ltd., Hungary). The “Membrane IHC Quantification” module based on the HALO image analysis platform was then utilized to identify and enumerate the total cells and CD8^+^ TILs within the defined tumor regions, and the area of each region was measured. CD8^+^ T lymphocytes were quantified according to the percentage and density of CD8^+^ TILs. The CD8^+^ TIL percentage (%) was determined as the ratio of CD8^+^ TILs to total cells, and the CD8^+^ TIL density (cells/mm^2^) was computed by dividing the total count of positively stained T cells by the total compartment area.

### 4.9 Immunotherapy Cohort

To preliminarily explore the association between immune phenotypes and therapeutic efficacy, we established a dedicated immunotherapy cohort. Through systematic retrospective review across participating centers from 2016 to 2024, we identified 20 IMA patients who received ICI therapy. Among them, 14 patients had undergone prior surgical resection and subsequently received ICI therapy at the time of relapse or metastasis. The remaining six patients (P4, 12, 13, 14, 15, 20 in Figure S3) presented with stage IV metastatic disease at initial diagnosis, receiving ICI therapy as part of the treatment without surgical resection. Treatment regimens reflected real‐world clinical practice during the study period. Patients received various anti‐PD‐1 agents (e.g., Pembrolizumab, Tislelizumab, Camrelizumab, Sintilimab) with or in combination with chemotherapy or targeted therapy, consistent with contemporary treatment guidelines. Anti‐PD‐L1 agents (e.g., Atezolizumab) were also used. Treatments were administered as standard monotherapy (e.g., Pembrolizumab 200 mg every 3 weeks) or in combination regimens. The line of therapy included second‐line and later settings.

### 4.10 Single‐Cell RNA Sequencing Analysis

To further characterize the immune microenvironment of IMA at a transcriptional level, we performed an integrated analysis of single‐cell RNA sequencing data. Data from eight lung adenocarcinoma samples (IMA: *n* = 2; NMA: *n* = 6) were obtained from two public datasets [28, 29]. Raw gene expression matrices were processed using the Seurat R package. Cells with unique feature counts less than 200 or greater than 6000, or with mitochondrial gene content exceeding 15%, were filtered out. Data normalization, scaling, and identification of highly variable genes were performed sequentially. Principal component analysis (PCA) was conducted on scaled data, and significant principal components were selected for graph‐based clustering and uniform manifold approximation and projection (UMAP) for dimensionality reduction.

Major cell lineages were annotated based on the expression of canonical marker genes, with reference to the CellMarker 2.0 database [30]. For subsequent focused analysis, T and NK cells were re‐clustered using an identical workflow to identify finer immune subsets. The relative proportions of effector CD8^+^ T cells were compared between the IMA and NMA groups. Furthermore, the expression level of PD‐1 within the effector CD8^+^ T cell compartment was quantified and compared across groups.

### 4.11 Statistical Analysis

Stata 16.0 and R 4.2.1 were used for statistical analysis and visualization. Associations between PD‐L1 expression, CD8^+^ TIL infiltration, and clinicopathological characteristics were assessed by chi‐squared or Fisher's exact tests, as appropriate. Statistical comparisons of the density and percentage of CD8^+^ TILs between PD‐L1‐positive and PD‐L1‐negative patients were conducted using either the Mann‒Whitney *U* test or Student's *t*‐test, depending on appropriateness. Scatter plots were constructed, and Spearman's test was employed to assess the linear correlation between the percentage and density of CD8^+^ TILs. The percentages of patients who were PD‐L1‐positive among the three groups with different CD8^+^ TIL infiltration levels were compared, and the Cochran–Armitage test was utilized to test the trends. X‐tile 3.6.1, a software from Yale University [31], was utilized to ascertain the optimal cutoff values for the percentage and density of CD8^+^ TILs for predicting RFS and OS. Kaplan–Meier plots and log‐rank tests were conducted to calculate the probabilities of OS and RFS. Subgroup analyses were performed based on adjuvant therapy status, with results presenting as forest plots. Univariate and multivariate Cox models identified independent prognostic factors. When constructing the multivariate models, we considered not only the clinical significance and statistical results of each variable in univariate analyses but also the potential multicollinearity between variables. A variance inflation factor (VIF) [32] greater than 5 was considered indicative of multicollinearity in the model. *p*‐values (two‐sided) less than 0.05 were defined as statistically significant.

## Author Contributions

Conceptualization: Guochao Zhang, Chao Zheng; Qi Xue; Data curation: Chao Zheng, Jia Jia, Lide Wang, Long Zhang, Yuzhuo Zhang, Meng Yue, Shuangping Zhang, Yuuping Liu, Liyan Xue; Formal analysis: Guochao Zhang, Chao Zheng, Jia Jia, Xingchen Li; Funding acquisition: Guochao Zhang, Qi Xue, Jie He; Investigation: Chao Zheng, Jia Jia, Lide Wang, Long Zhang, Yuzhuo Zhang, Meng Yue, Shuangping Zhang, Yuuping Liu, Liyan Xue; Methodology: Guochao Zhang, Chao Zheng; Project administration: Liyan Xue, Qi Xue, Jie He; Resources: Liyan Xue, Qi Xue, Jie He; Software: Guochao Zhang, Chao Zheng, Jia Jia, Xingchen Li; Supervision: Liyan Xue, Qi Xue, Jie He; Validation: Guochao Zhang, Chao Zheng, Jia Jia, Xingchen Li; Visualization: Guochao Zhang, Chao Zheng, Jia Jia, Xingchen Li; Writing – original draft: Guochao Zhang, Chao Zheng, Jia Jia, Xingchen Li; Writing – review & editing: All authors. All authors have read and approved the final manuscript.

## Funding Information

This work was supported by the CAMS Innovation Fund for Medical Sciences (CIFMS) (2025‐12M‐XHXX‐051; 2025‐I2M‐C&T‐B‐058; 2025‐I2M‐C&T‐B‐011); Non‐profit Central Research Institute Fund of Chinese Academy of Medical Sciences (2025‐JKCS‐30); National High Level Hospital Clinical Research Funding and Cooperation Fund of CHCAMS Beijing & Langfang & SZCH (CFA202501002; CFA202503003); CAMS Initiative for Innovative Medicine (2021‐1‐I2M−012); National High Level Hospital Clinical Research Funding (LC2024D01, 8010202).

## Ethics Statement

This study was approved by the ethics committees of the National Cancer Center (ethics number: 22/244‐3446), with the written informed consent from patients waived.

## Conflicts of Interest

The authors declare no conflicts of interest.

## Supporting information




**Supporting File 1**: mco270710‐sup‐0001‐SupMat.docx

## Data Availability

The single‐cell RNA sequencing data analyzed in this study are derived from two public sources: the Gene Expression Omnibus dataset GSE223923 and the dataset available at https://codeocean.com/capsule/8321305/tree/v1. All other data generated during this study are available upon reasonable request to the corresponding author (Prof. Jie He, Qi Xue, and Liyan Xue).
