# Supplementary material for: Prognostic Implications of Programmed Cell Death Ligand 1 Expression, Cluster of Differentiation 8‐Positive T‐Cell Infiltration, and Related Immunophenotypes in Invasive Mucinous Adenocarcinoma of the Lung: A Multicenter Study
Source: MedComm (2020). 2026 Apr 5;7(4):e70710. doi: 10.1002/mco2.70710 (PMC13051811; doi:10.1002/mco2.70710)
Supplement: Supplementary file 1 — Supporting File 1: mco270710‐sup‐0001‐SupMat.docx [file MCO2-7-e70710-s001.docx]

**Supplementary Materials for**

**Prognostic Implications of PD-L1 Expression, CD8+ T-Cell Infiltration, and Related Immunophenotypes in Invasive Mucinous Adenocarcinoma of the Lung: A Multicenter Study**

Guochao Zhang^1, #^, Chao Zheng^1, 2, #,^ Jia Jia^3, #^, Xingchen Li^1, #^, Lide Wang^1^, Long Zhang^1^, Yuzhuo Zhang^1^, Meng Yue^4^, Shuangping Zhang^5^, Yueping Liu^4^, Liyan Xue^3, *^, Qi Xue^1, *^, Jie He^1, *^

^1^Department of Thoracic Surgery, National Cancer Center/National Clinical Research Center for Cancer/Cancer Hospital, Chinese Academy of Medical Sciences and Peking Union Medical College, Beijing, China, 100021;

^2^Department of Cancer Prevention, National Cancer Center/National Clinical Research Center for Cancer/Cancer Hospital, Chinese Academy of Medical Sciences and Peking Union Medical College, Beijing, China, 100021;

^3^Department of Pathology, National Cancer Center/ National Clinical Research Center for Cancer/Cancer Hospital, Chinese Academy of Medical Sciences and Peking Union Medical College, Beijing, China, 100021;

^4^Department of Pathology, Fourth Hospital of Hebei Medical University/Tumor Hospital of Hebei Province, Shijiazhuang, Hebei, China, 050000;

^5^Department of Thoracic Surgery, Shanxi Province Cancer Hospital/Shanxi Hospital Affiliated to Cancer Hospital, Chinese Academy of Medical Sciences, Taiyuan, Shanxi, China, 030013.

^#^These authors contributed equally.

***Corresponding author:**

**Prof. Jie He:** Department of Thoracic Surgery, National Cancer Center/ National Clinical Research Center for Cancer/Cancer Hospital, Chinese Academy of Medical Sciences and Peking Union Medical College, Beijing, 100021, China; e-mail: profjiehe@126.com;

**Prof. Qi Xue:** Department of Thoracic Surgery, National Cancer Center/ National Clinical Research Center for Cancer/Cancer Hospital, Chinese Academy of Medical Sciences and Peking Union Medical College, Beijing, 100021, China; e-mail: xueqi@cicams.ac.cn;

**Prof. Liyan Xue:** Department of Pathology, National Cancer Center/National Clinical Research Center for Cancer/Cancer Hospital, Chinese Academy of Medical Sciences and Peking Union Medical College, Beijing, 100021, China; e-mail: xuely@cicams.ac.cn;

**This PDF file includes** Figure S1–4 and Tables. S1–5.


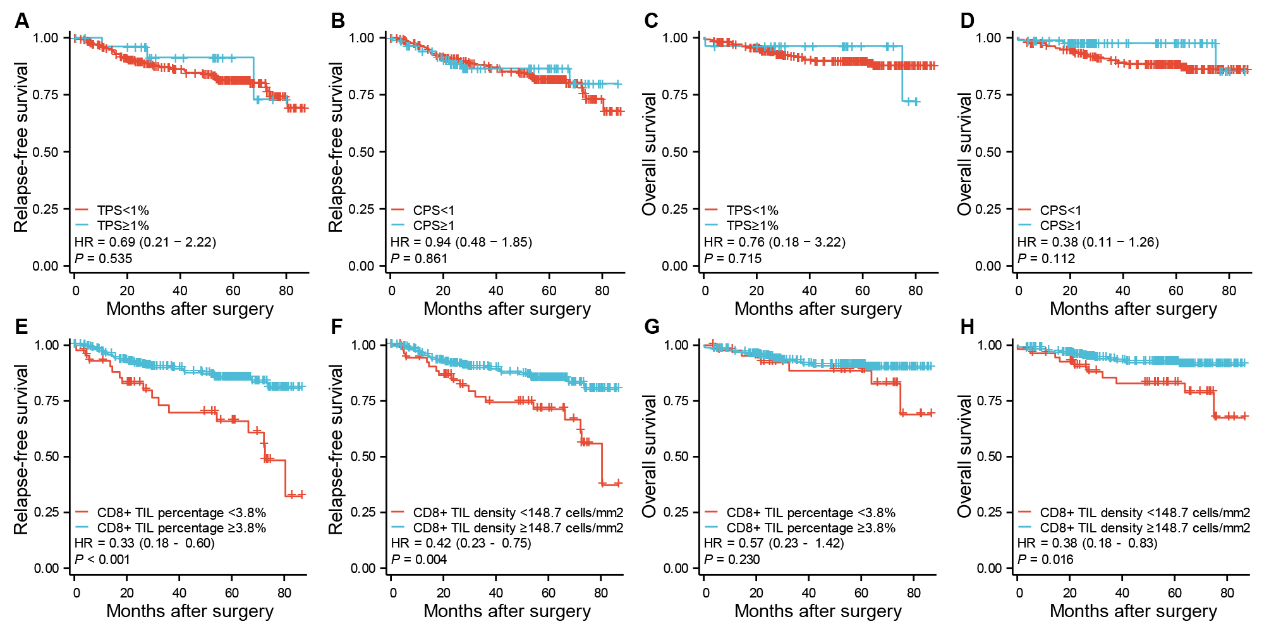


**Figure S1.** Prognostic significance of PD-L1 expression and CD8+ TIL infiltration. Kaplan-Meier survival curves in IMA patients. (A) Relapse-free survival (RFS) stratified by PD-L1 TPS. (B) RFS stratified by PD-L1 CPS. (C) Overall survival (OS) stratified by PD-L1 TPS. (D) OS stratified by PD-L1 CPS. (E) RFS stratified by CD8+ TIL percentage. (F) RFS stratified by CD8+ TIL density. (G) OS stratified by CD8+ TIL percentage. (H) OS stratified by CD8+ TIL density.


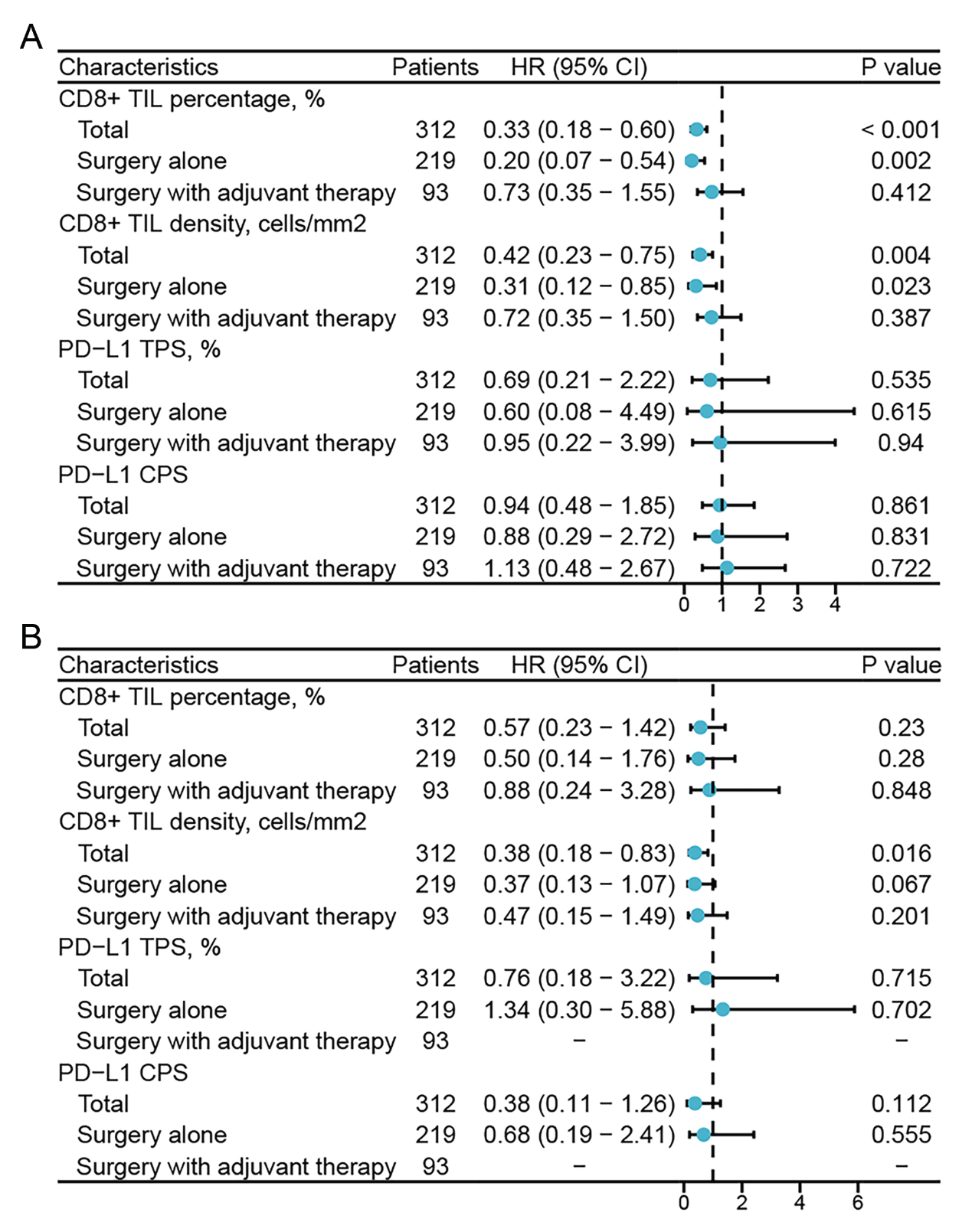


**Figure S2.** Forest plots of prognostic associations for CD8⁺ TILs and PD-L1 stratified by adjuvant therapy. (A) Hazard ratios for Relapse-Free Survival (RFS). (B) Hazard ratios for Overall Survival (OS). In both panels, patients are stratified by receipt of adjuvant therapy. Each row represents a biomarker variable (CD8⁺ TIL percentage, CD8⁺ TIL density, PD-L1 TPS, PD-L1 CPS).


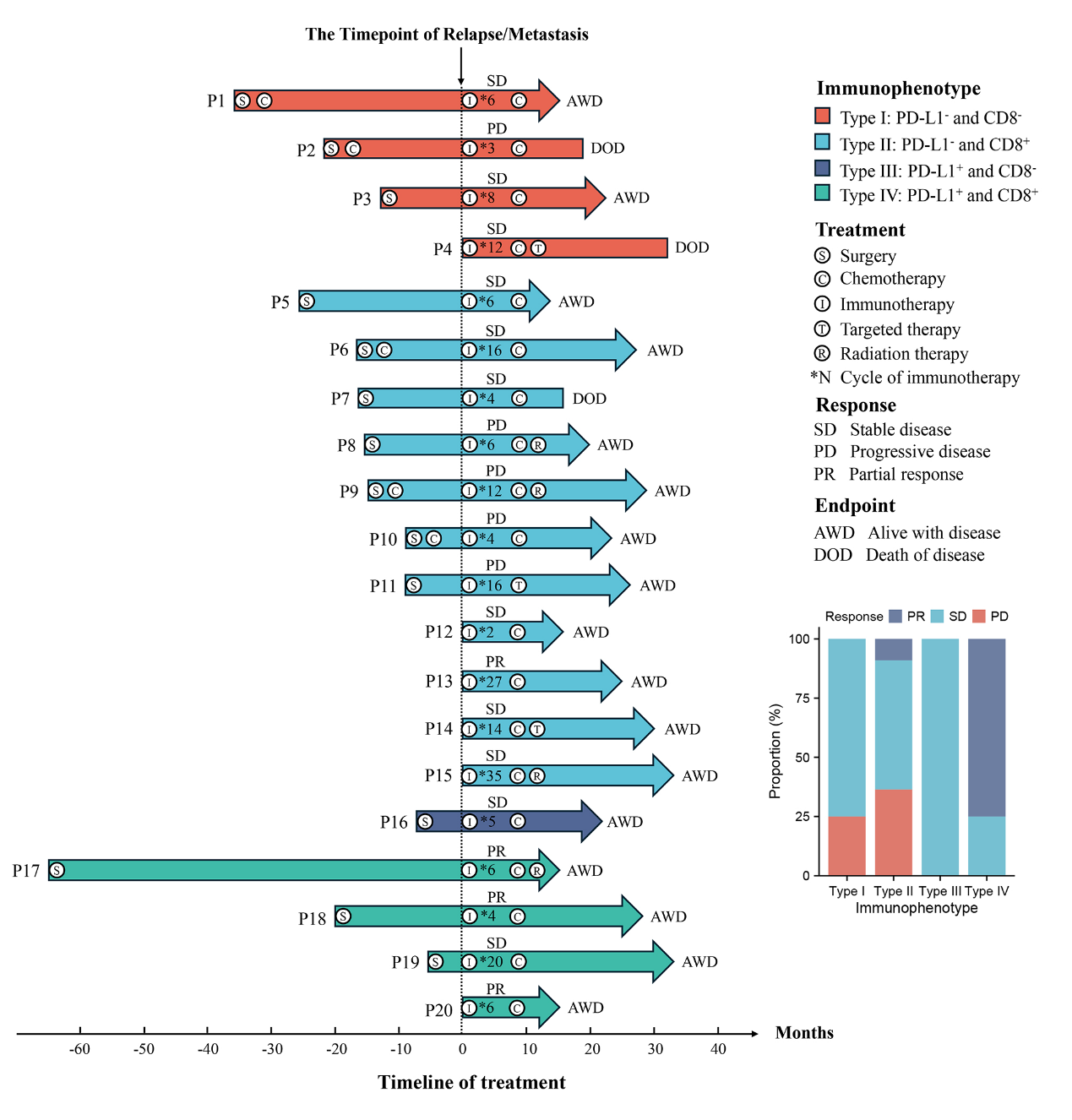


**Figure S3.** The treatment timeline, number of immunotherapy cycles, and best treatment response of 20 IMA patients who received immunotherapy


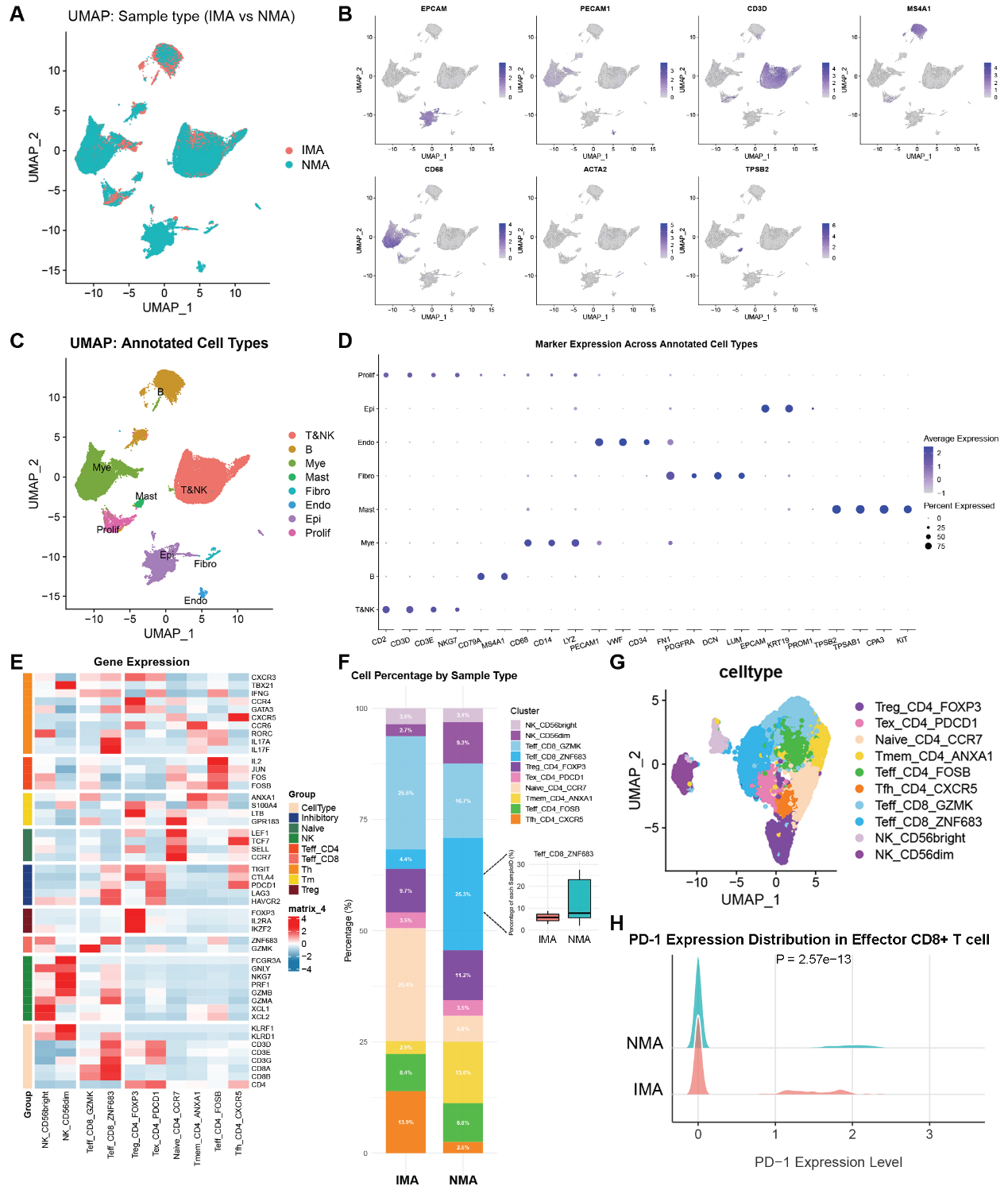


**Figure S4.** Single-cell profiling reveals an attenuated effector CD8⁺ T cell in IMA. (A) UMAP visualization of cells from IMA (n=2) and NMA (n=6) samples. (B) Expression of canonical lineage marker genes. (C) Annotated major cell types. (D) Dot plot of marker gene expression across cell types. (E) Heatmap of marker genes for T/NK subsets. (F) Proportional composition of T/NK subsets, showing a significantly lower proportion of the Teff_CD8_ZNF683 subset in IMA (4.4%) versus NMA (25.3%). (G) UMAP of annotated T/NK subsets. (H) Violin plots show significantly lower PD-1 expression in IMA effector T cells (p = 2.57e-13).

**Table S1**. Baseline characteristics of 312 patients with invasive mucinous adenocarcinoma of the lung

| **Characteristic** | **No. of patients**  **Total (N=312)** | **%** |
| --- | --- | --- |
| Gender |  |  |
| Male | 125 | 40.1 |
| Female | 187 | 59.9 |
| Age, y |  |  |
| ≤60 | 159 | 51.0 |
| >60 | 153 | 49.0 |
| Alcohol abuse |  |  |
| Yes | 53 | 17.0 |
| No | 259 | 83.0 |
| Smoking |  |  |
| Yes | 83 | 26.6 |
| No | 229 | 73.4 |
| Smoking index |  |  |
| ≤400 | 263 | 84.3 |
| >400 | 49 | 15.7 |
| Family history of malignant tumor |  |  |
| Yes | 51 | 16.3 |
| No | 261 | 83.7 |
| Comorbidities |  |  |
| Yes | 133 | 42.6 |
| No | 179 | 57.4 |
| Treatment modality |  |  |
| Surgery alone | 219 | 70.2 |
| Surgery with adjuvant therapy | 93 | 29.8 |
| Surgical approach |  |  |
| VATS | 276 | 88.5 |
| Open | 36 | 11.5 |
| Laterality |  |  |
| Left | 155 | 49.7 |
| Right | 157 | 50.3 |
| Lobe |  |  |
| Upper | 76 | 24.4 |
| Middle | 16 | 5.1 |
| Lower | 213 | 68.3 |
| Overlapping | 7 | 2.2 |
| Tumor Size, cm |  |  |
| ≤3 | 193 | 61.9 |
| >3 | 119 | 38.1 |
| Genetic Test |  |  |
| NA | 116 | 37.2 |
| None | 82 | 26.3 |
| KRAS mutation | 85 | 27.2 |
| EGFR mutation | 13 | 4.2 |
| Others | 16 | 5.1 |
| T stage |  |  |
| T1 | 176 | 56.4 |
| T2 | 86 | 27.6 |
| T3 | 24 | 7.7 |
| T4 | 26 | 8.3 |
| N stage |  |  |
| N0 | 278 | 89.1 |
| N1 | 8 | 2.6 |
| N2 | 26 | 8.3 |
| TNM stage |  |  |
| I | 216 | 69.2 |
| II | 45 | 14.4 |
| III | 51 | 16.4 |
| PD-L1 expression by TPS |  |  |
| <1% (negative) | 284 | 91.0 |
| ≥1% (positive) | 28 | 9.0 |
| PD-L1 expression by CPS |  |  |
| <1 (negative) | 223 | 71.5 |
| ≥1 (positive) | 89 | 28.5 |
| CD8+ TIL density, mm^2^ |  |  |
| Median | 320.2 |  |
| Range | 20.6–1912.1 |  |
| <148.7 cells/mm2 (negative) | 56 | 18.0 |
| ≥148.7 cells/mm2 (positive) | 256 | 82.0 |
| CD8+ TIL percentage, % |  |  |
| Median | 8.3 |  |
| Range | 0.5–34.5 |  |
| <3.8% (negative) | 44 | 14.1 |
| ≥3.8% (positive) | 268 | 85.9 |
| **Abbreviations:** VATS, video-assisted thoracoscopic surgery; NA, not applicable; TNM, tumor node metastasis stage; TPS, tumor positive score; CPS, combined positive score. | | |

**Table S2.** The detailed grouping information of 4 combinations of PD-L1 and CD8 evaluation criteria.

| **PD-L1** | **CD8** | **Patient number (Total N=218)** | | | |
| --- | --- | --- | --- | --- | --- |
|  |  | **Type I (-/-)** | **Type II (-/+)** | **Type III (+/-)** | **Type IV (+/+)** |
| TPS score | CD8+ TIL percentage | 41 | 243 | 3 | 25 |
| TPS score | CD8+ TIL density | 53 | 231 | 3 | 25 |
| CPS score | CD8+ TIL percentage | 36 | 187 | 8 | 81 |
| CPS score | CD8+ TIL density | 47 | 176 | 9 | 80 |

**Abbreviations:** TPS, tumor positive score; CPS, combined positive score.

**Notes:** Positive (+) cutoff values for TPS, CPS, CD8+ TIL density, CD8+ TIL percentage were ≥1%, ≥1, ≥148.7 cells/mm^2^, ≥3.8%, respectively; Negative (-) cutoff values for TPS, CPS, CD8+ TIL density, CD8+ TIL percentage were <1%, <1, <148.7 cells/mm^2^, <3.8%, respectively.

**Table S3.** Univariate Cox regression analysis of PD-L1 expression, CD8 infiltration, and clinicopathological characteristics in IMA patients.

| Variable |  | Relapse-free survival |  | Overall survival |  |
| --- | --- | --- | --- | --- | --- |
|  |  | HR (95% CI) | *P* value | HR (95% CI) | *P* value |
| Gender | Male vs. Female | 3.05 (1.72–5.40) | <0.001 | 3.55 (1.61–7.86) | 0.002 |
| Age | >60 vs. ≤60 | 0.69 (0.40–1.23) | 0.209 | 0.93 (0.44–1.95) | 0.843 |
| Alcohol abuse | Yes vs. No | 1.90 (1.02–3.53) | 0.042 | 2.97 (1.38–6.35) | 0.005 |
| Smoking | Yes vs. No | 2.72 (1.56–4.75) | <0.001 | 3.82 (1.81–8.09) | <0.001 |
| Family history of malignant tumor | Yes vs. No | 1.45 (0.72–2.89) | 0.297 | 0.69 (0.21–2.29) | 5.545 |
| Comorbidities | Yes vs. No | 0.65 (0.36–1.16) | 0.145 | 1.11 (0.53–2.34) | 0.780 |
| Treatment modality | Surgery with adjuvant therapy vs. Surgery alone | 4.83 (2.68–8.70) | <0.001 | 1.68 (0.79–3.56) | 0.175 |
| Surgical approach | VATS vs. Open | 0.59 (0.29–1.18) | 0.132 | 0.31 (0.15–0.70) | 0.004 |
| Laterality | Right vs. Left | 1.26 (0.72–2.20) | 0.422 | 1.14 (0.54–2.38) | 0.738 |
| Lobe | Upper, Middle, Overlapping vs. Lower | 1.81 (1.04–3.17) | 0.037 | 1.64 (0.78–3.48) | 0.191 |
| Tumor Size, cm | >3 vs. ≤3 | 3.49 (1.96–6.23) | <0.001 | 4.34 (1.91–9.87) | <0.001 |
| Genetic Test | KRAS/EGFR/Others vs. None | 1.23 (0.85–1.80) | 0.230 | 0.38 (0.13–1.05) | 0.062 |
| T stage | T3-4 vs. T1-2 | 3.72 (2.08–6.77) | <0.001 | 5.25 (2.49–11.06) | <0.001 |
| N stage | N1-3 vs. N0 | 5.14 (2.79–9.47) | <0.001 | 4.62 (2.08–10.28) | <0.001 |
| TNM stage | II vs. I | 3.00 (1.39–6.46) | 0.005 | 3.10 (1.13–8.56) | 0.029 |
|  | III vs. I | 7.34 (3.92–13.75) | <0.001 | 5.91 (2.55–13.72) | <0.001 |
| PD-L1 expression by TPS | Positive vs. Negative | 0.69 (0.21–2.22) | 0.535 | 0.76 (0.18–3.22) | 0.715 |
| PD-L1 expression by CPS | Positive vs. Negative | 0.94 (0.48–1.85) | 0.861 | 0.38 (0.11–1.26) | 0.112 |
| CD8+ TIL density, mm^2^ | Positive vs. Negative | 0.42 (0.23–0.75) | 0.004 | 0.38 (0.18–0.83) | 0.016 |
| CD8+ TIL percentage, % | Positive vs. Negative | 0.33 (0.18–0.60) | <0.001 | 0.57 (0.23–1.42) | 0.230 |
| **Abbreviations:** HR, hazard ratio; CI, confidence interval; VATS, video-assisted thoracoscopic surgery; NA, not applicable; TNM, tumor node metastasis stage; TPS, tumor positive score; CPS, combined positive score. | | | | | |

**Table S4**. Distribution of 312 patients with invasive mucinous adenocarcinoma of the lung from three hospitals in China.

| **Hospital** | **City, Province** | **Patient number** |
| --- | --- | --- |
| National Cancer Center/National Clinical Research Center for Cancer/Cancer Hospital, Chinese Academy of Medical Sciences and Peking Union Medical College | Beijing, Beijing | 223 |
| Fourth Hospital of Hebei Medical University/Tumor Hospital of Hebei Province | Shijiazhuang, Hebei | 83 |
| Shanxi Province Cancer Hospital/Shanxi Hospital Affiliated to Cancer Hospital, Chinese Academy of Medical Sciences | Taiyuan, Shanxi | 7 |

**Table S5.** The detailed information of main reagents used in the study.

| **Reagents** | **Clone** | **Manufacturer** | **Country of origin** |
| --- | --- | --- | --- |
| Hydrogen peroxide (H_2_O_2_) | - | Dako | Glostrup, Denmark |
| 3,3′-diaminobenzidine (DAB) | - | Dako | Glostrup, Denmark |
| Rabbit monoclonal anti-PD-L1 | clone E1L3N | Amoy Diagnostics | Xiamen, China |
| Mouse monoclonal anti-CD8 | clone C8/144B | Dako | Carpinteria, CA, USA |
| Rabbit monoclonal anti-CD4 | clone 458G4A1 | Abcarta | Suzhou, China |
| Rabbit monoclonal anti-CD8 | clone 815R4B2 | Abcarta | Suzhou, China |
| Cytokeratin-pan | Clone AE1/AE3 | MXB Biotechnologies | Fuzhou, China |
| goat anti-rabbit-HRP | - | Dako | Glostrup, Denmark |
| goat anti-mouse-HRP | - | Dako | Glostrup, Denmark |
